# Interactions between Glucocorticoid Treatment and Cis-Regulatory Polymorphisms Contribute to Cellular Response Phenotypes

**DOI:** 10.1371/journal.pgen.1002162

**Published:** 2011-07-07

**Authors:** Joseph C. Maranville, Francesca Luca, Allison L. Richards, Xiaoquan Wen, David B. Witonsky, Shaneen Baxter, Matthew Stephens, Anna Di Rienzo

**Affiliations:** 1 Department of Human Genetics, The University of Chicago, Chicago, Illinois, United States of America; 2 Department of Statistics, The University of Chicago, Chicago, Illinois, United States of America; Georgia Institute of Technology, United States of America

## Abstract

Glucocorticoids (GCs) mediate physiological responses to environmental stress and are commonly used as pharmaceuticals. GCs act primarily through the GC receptor (GR, a transcription factor). Despite their clear biomedical importance, little is known about the genetic architecture of variation in GC response. Here we provide an initial assessment of variability in the cellular response to GC treatment by profiling gene expression and protein secretion in 114 EBV-transformed B lymphocytes of African and European ancestry. We found that genetic variation affects the response of nearby genes and exhibits distinctive patterns of genotype-treatment interactions, with genotypic effects evident in either only GC-treated or only control-treated conditions. Using a novel statistical framework, we identified interactions that influence the expression of 26 genes known to play central roles in GC-related pathways (e.g. *NQO1*, *AIRE*, and *SGK1*) and that influence the secretion of IL6.

## Author Summary

Glucocorticoids (GCs) are steroid hormones produced by the human body in response to environmental stressors. Despite their key role as physiological regulators and widely administered pharmaceuticals, little is known about the genetic basis of inter-individual and inter-ethnic variation in GC response. As GC action is mediated by the regulation of gene expression, we profiled transcript abundance and protein secretion in EBV-transformed B lymphocytes from a panel of 114 individuals, including those of both African and European ancestry. Combining these molecular traits with genome-wide genetic data, we found that genotype-treatment interactions at polymorphisms near genes affected GC regulation of expression for 26 genes and of secretion for IL6. A novel statistical approach revealed that these interactions could be distinguished into distinct types, with some showing genotypic effects only in GC-treated samples and others showing genotypic effects only in control-treated samples, with differing phenotypic and molecular interpretations. The insights into the genetic basis of variation in GC response and the statistical tools for identifying gene-treatment interactions that we provide will aid future efforts to identify genetic predictors of response to this and other treatments.

## Introduction

Glucocorticoids (GCs) are steroid hormones that mediate homeostatic responses to environmental stressors through the regulation of critical physiological processes (e.g. immune response, energy metabolism and blood pressure (reviewed in [1])). Owing to early observations of the anti-inflammatory properties [2] of cortisol (i.e. the endogenous GC in humans), synthetic GCs are widely used as pharmaceuticals for inflammatory and autoimmune diseases (e.g. asthma [3] and rheumatoid arthritis [4]). GCs are also used in the treatment of several types of cancer [5], most notably lymphoid malignancies [6], due to their pro-apoptotic activities and for symptomatic relief. While there is evidence for a substantial genetic contribution [7]–[12], and for inter-ethnic differences in drug response [13], [14], little is known about the genetic architecture of variation in GC response within and between human populations.

Genetic effects on GC action could provide a mechanism for a vast array of gene-environment interactions, which could have major implications for human phenotypic variation. In fact, evidence of such interactions has been observed for numerous traits relevant to GCs including obesity [15], cardiovascular disease [16] and asthma [17]. With few exceptions (e.g. a regulatory polymorphism in the promoter of *IL6* [18]), little is currently known about the mechanisms that underlie gene-environment interactions. If not properly accounted for, these interactions can complicate efforts to identify genetic and environmental factors associated with disease risk. Furthermore, identifying genetic variation that interacts with pharmaceutical treatments like GCs, which are a specific subset of environmental factors, is of particular interest from a clinical perspective and constitutes the primary goal of pharmacogenetics.

As GCs act largely by inducing changes in the expression of target genes [19], regulatory polymorphisms are likely to contribute to variation in response. The initial steps of the GC response pathway are mediated by the GC receptor (GR) and interacting transcription factors. GC binding allows the GR to translocate from the cytoplasm to the nucleus, where it regulates gene expression through at least two distinct mechanisms. The GR can either drive the assembly of novel transcriptional regulatory complexes at target genes, or inhibit regulatory complexes, such as NFκB [20], that are already active at target genes. Some direct GR target genes are, in turn, transcription factors that regulate downstream target genes.

Here, we provide an initial view of the genetic architecture of variation in the GC-mediated regulation of transcription and protein secretion. To accomplish this, we measured the expression of 13,232 genes and the secretion levels of 10 proteins in paired aliquots, one treated with the synthetic GC dexamethasone (dex) and one treated with the vehicle for dex (EtOH) as a control, in a panel of 114 densely genotyped HapMap B-lymphocytes transformed with Epstein-Barr Virus (EBV), commonly known as lymphoblastoid cell lines (LCLs). This panel included 57 Yoruba (YRI) from Nigeria and 57 Toscani (TSI) from Italy. EBV transformation proceeds, in part, by mimicking CD40 activation and ultimately leads to cellular proliferation through a variety of mechanisms, including the activation of the NFκB signaling pathway [21]. Given their activated state, LCLs are a suitable system for studying the immunorepressive effects of GCs. Additionally, some regulatory variants that affect GC response in LCLs may be shared with other cell types, as observed for baseline expression [22]–[24].

## Results

### GCs have widespread effects on transcriptional regulation

We found that 4,568 genes were differentially expressed, at a FDR<0.01 (p<0.003), following treatment with GCs (8 h, 1 uM dexamethasone), corresponding to ∼32% of the expressed genes. This number is similar to that observed in a recent study of equivalent sample size in osteoblasts treated with GCs [25], but larger than previous studies that used much smaller samples (often a single cell line; e.g. [26]). This suggests that large sample sizes are necessary to identify many GC target genes. Accordingly, we found that sub-sampling data from our full panel of LCLs dramatically reduced the number of differentially expressed genes, especially at genes with inter-individual variation in transcriptional response (Figure S1). It should be noted that tests of differential expression rely on magnitude of transcriptional response and its consistency across individuals. Because our main goal is to identify the genetic basis of variation in response, we did not limit our mapping analyses (see below) to the differentially expressed genes.

Among the differentially expressed genes in LCLs, we found roughly equal numbers of up and down-regulated genes. Up-regulated genes were enriched for GC-related biological processes including cellular response to stimulus (p = 4.1×10^−6^, FDR = 7.5×10^−5^) and cell cycle (p = 1.4×10^−5^, FDR = 2.6×10^−4^), consistent with GC regulation of lymphocyte proliferation. Down-regulated genes were enriched for immune response genes (p = 1.1×10^−10^, FDR = 4.6×10^−9^) and for genes involved in the positive regulation of I-kappaB kinase/NF-kappaB cascade (p = 3.3×10^−5^, FDR = 3.3×10^−4^), consistent with the immunorepressive role of GCs.

To explore the extent of tissue-specificity in the transcriptional response to GCs, we compared our data to the results in osteoblasts [25]. We found a significant overlap between the genes differentially expressed in LCLs and in osteoblasts (p = 4.8×10^−13^), but only 28% of genes differentially expressed in our study are differentially expressed (p<0.05) in osteoblasts. This likely reflects some amount of tissue specificity, although other factors are likely to contribute (e.g. incomplete power [23], differences in duration of treatment).

We measured and corrected for multiple factors related to EBV-transformation that have been previously shown to be associated with gene expression patterns at baseline [27], [28] (e.g. EBV copy number). Unlike baseline expression, these factors showed hardly any evidence for an effect on transcriptional response (see Table S1); nonetheless, we corrected for them in all subsequent analyses.

### No evidence for trans-acting genetic effects on transcriptional response to GCs

**Figure 1:**
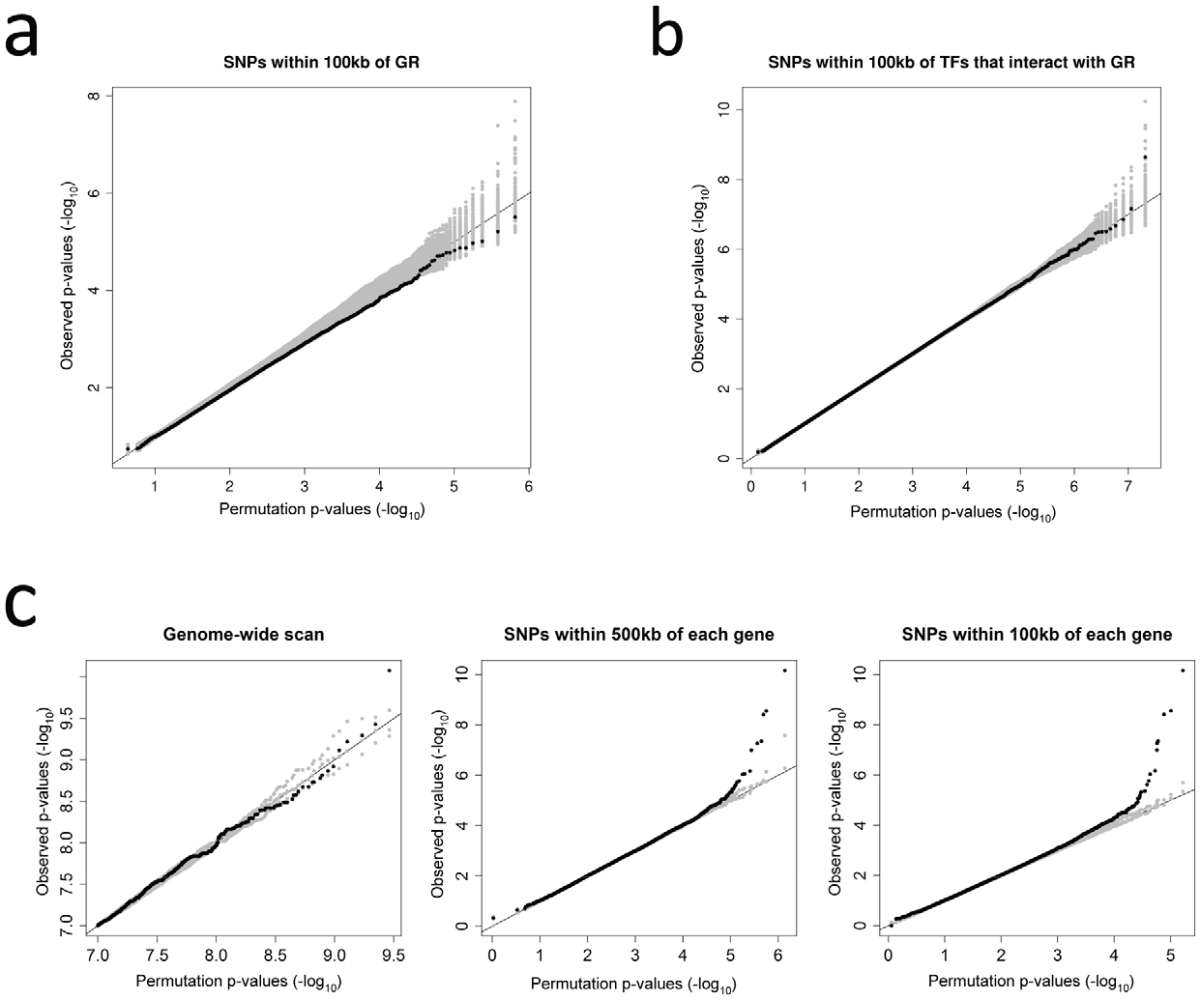
Quantile-quantile plots summarizing the results from tests for genetic variation associated with log fold change in expression. a) Test of all HapMap SNPs within 100 kb of the gene that encodes GR. b) Test of all HapMap SNPs within transcription factors that interact with the GR. c) Results from genome-wide scan (all HapMap SNPs) are compared to scan limited to SNPs within 500 kb and 100 kb of each gene. Observed p-values are shown as black dots. P-values from permutations are shown as grey dots. Results from 100 permutations are shown for a) and b), and results from 3 permutations are shown for c).

Many of the proteins involved in the GC-mediated regulation of transcription are well characterized (i.e. GR and interacting transcription factors). Genetic variants that impact the function of these regulatory proteins are likely to influence transcriptional response at several, and potentially many, downstream genes. Consequently, the genes that encode these proteins are candidate expression quantitative trait loci (eQTLs) acting in *trans* to modulate the transcriptional response to GCs. However, genome-wide tests for *trans* eQTLs suffer from a tremendous multiple testing burden. Therefore, to reduce the number of tests being performed, we first examined only these candidate genes for response eQTLs. We used simple linear regression to test for an association between log fold change in expression at each expressed gene in the genome and genotype at all HapMap SNPs within 100 kb of the gene that encodes the GR (*NR3C1*), and found no significant evidence of association at a FDR<0.2 (Figure 1a, Figure S4a-S4b). Similarly, as the GR interacts with other transcription factors in the regulation of target gene transcription, we also tested all HapMap SNPs within 100 kb of 34 genes that encode transcription factors known to interact with the GR (listed in Materials and Methods [29]). Here again, we found no evidence for an effect of genetic variation at these loci on the transcriptional response to GCs at a FDR<0.2 (Figure 1b, Figure S4c-S4d).

### Evidence for local effects on transcriptional response to GCs

**Figure 2:**
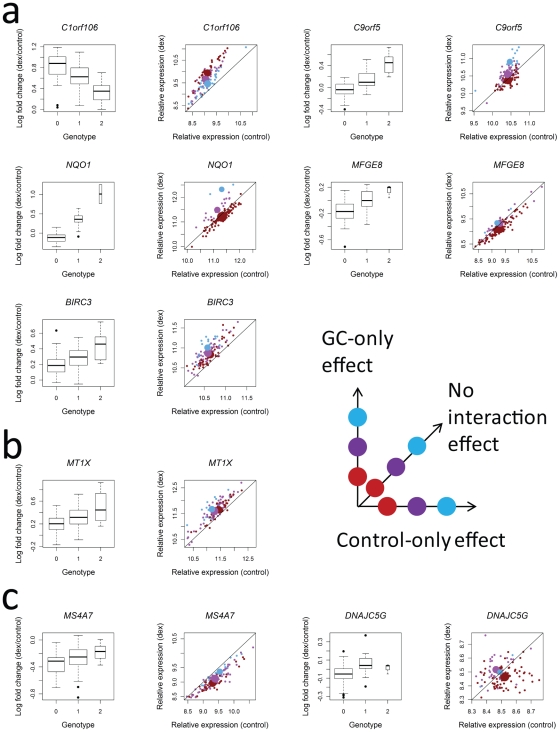
Patterns of interaction between genotype and GC treatment that underlie associations with log fold change for each of the 8 genes where log fold change is significantly associated with genotype at a SNP within 100 kb. Plots on the left show the effect of genotype on log_2_ fold change, with genotype coded as copies of the minor allele. In plots on the right, each small dot corresponds to an individual and is color coded based on genotype (red = homozygous for major allele, purple = heterozygous, and blue = homozygous for minor allele). Large dots represent genotypic means. Associations are classified based on the configuration of genotypic effects in the two conditions, including a) genotypic effects only in GC-treated samples, b) genotypic effects only in control-treated samples and c) genotypic effects in both conditions that differ. On the right is a cartoon showing patterns of interaction in two-dimensional space with expression after GC-treatment on the y-axis and expression after control-treatment on the x-axis. Each of the three dots on each line corresponds to the mean value for a genotype class (red = homozygous for major allele, purple = heterozygous, and blue = homozygous for minor allele). Genotype is coded as copies of the minor allele. Relative expression corresponds to log_2_-transformed microarray intensities.

We then performed an unbiased, genome-wide scan for genetic variation associated with GC response. Specifically, we tested for an association between every HapMap SNP and log fold change at every gene. While this analysis did not reveal any significant associations at a FDR<0.2, we found that the top association was between log fold change at *C1orf106* and genotype at an intronic SNP (rs4915463, p = 8.4×10^−11^, FDR<0.67). Given the proximity of the associated SNP to the *C1orf106* locus and work by others highlighting the impact of cis-acting regulatory polymorphisms on baseline expression [30], [31], we then focused our analyses on HapMap SNPs near each of the 12,619 expressed, autosomal genes. We found the strongest signal when we tested for an association between log fold change at each gene and all SNPs within 100 kb (compared to either a genome-wide scan or testing SNPs within 500 kb of each gene, Figure 1c). This analysis revealed local response eQTLs for 8 genes at a FDR<0.1 (Figure 2). These included genes previously shown to play important roles in GC-related biological processes, including regulation of immune response (*MT1X* [32] and *MFGE8* [33]) and cell cycle progression (e.g. *BIRC3* [34]). These also included *NQO1*, a gene previously shown to affect variation in response to GC pharmaceutical treatment [35].

### Distinguishing types of genotype-treatment interactions that influence response

Visual examination of the genes in Figure 2 indicates that different genes show qualitatively different patterns. For some genes, a genotypic effect is evident either in only the GC-treated condition (*C1orf106, NQO1, C9orf5, MFGE8,* and *BIRC3*) or only the control-treated condition (*MT1X*). For others, an effect is evident in both, but differs between the two conditions (*DNAJC5G* and *MS4A7*). These different patterns may have different mechanistic and phenotypic interpretations, but are not distinguished by the test of log fold change, and so researchers have previously been forced to identify such patterns *post hoc* (e.g. [36]). To address this, we developed a novel statistical framework that explicitly compares and identifies these different patterns of interaction.

In brief, our method explicitly compares five different models relating each SNP to phenotypic measurements in the two treatment conditions (GC and control):

0. Null model: no association between genotype and phenotype in either condition.
1. No-interaction model: genotype is associated with phenotype in both conditions, with the same effect in each condition.
2. GC-only model: genotype is associated with phenotype in GC-treated samples, but not in control-treated samples.
3. Control-only model: genotype is associated with phenotype in control-treated samples, but not in GC-treated samples.
4. General interaction model: genotype is associated with phenotype in both conditions, but with different effects in each condition.

For each SNP, we computed a likelihood ratio, or Bayes Factor (BF) that measures the relative support in the data for each model 1–4. These BFs take account of the paired nature of the data, and the correlations between measurements in the same LCL in different conditions. We used a hierarchical model [37] to combine information both across SNPs in each gene region, and across genes, ultimately computing a posterior probability for each gene that it follows each of the models 1–4, i.e. that it is affected by a polymorphism that follows that model. We used these posterior probabilities both to identify high-confidence eQTLs of each type, and to estimate false discovery rates among eQTLs exceeding any given posterior probability threshold. This method is broadly applicable to the study of any gene-environment interactions with paired phenotype measurements.

Using this novel framework we identified 26 genes with high-confidence interactions (posterior probability of interaction>0.7, FDR = 0.10) between GC treatment and eQTLs. These interaction eQTLs included 7 of the 8 response eQTLs identified by mapping log fold change. The remainder generally showed strong, but not genome-wide significant (FDR<0.10), association with log fold change (see Table S2 and Figure S2). The larger number of interactions identified compared with mapping log fold change (26 versus 8), therefore, reflects an increase in power that comes from explicitly considering different plausible interaction scenarios. Of these, the majority (18 of 26) showed strongest support for GC-only interactions, with the remainder (8 of 26) showing strongest support for control-only eQTLs. Only one interaction between treatment and genotype identified through mapping the log fold change was not identified by the Bayesian hierarchical model (*DNAJC5G*). This interaction was the least significant of the 8 identified by mapping log fold change, and although it also shows some signal in the Bayesian analysis (BF for general interaction versus null = 4.9×10^2^, BF for general interaction versus no-interaction = 6.4×10^3^), the signal was insufficient to outweigh the low prior probability of a general interaction estimated by the hierarchical model (prior = 0.001, see Table S4).

The Bayesian hierarchical model revealed eQTLs at genes with clear biological relevance to GC-related biological processes that were not identified through mapping the log fold change. These include additional genes involved in the regulation of immune response (e.g. *CST7* [38] or *NLRP2* [39]) and cell cycle progression (e.g. *PDGFRL* [40]), well-established GC targets, such as serum and glucocorticoid regulated kinase 1 (*SGK1* [41]), and previously unknown GC target genes. For example, we found a control-only eQTL for multiple coagulation factor deficiency 2 (*MDCF2*), which is involved in the production of pro-coagulation factors [42]. The effect of GCs on coagulation is controversial [43], but has been suggested to play a role in their therapeutic effects on diseases such as asthma [44].

Given that cortisol regulates a variety of physiological processes relevant to numerous diseases, we compared our eQTL results to those from genome-wide association studies collected as a part of the GWAS catalog [45]. We found that a GC-only eQTL for *AIRE* (rs762421) was associated with risk of the Crohn's disease [46]. *AIRE* encodes a potent repressor of autoimmunity and can cause severe autoimmune disease when mutated [47]. In addition to its role in removing autoreactive T cells in the thymus, *AIRE* also plays a role in B-cell mediated immune response [48]. We found that the putative risk allele (rs762421-G) is associated with the down-regulation of *AIRE* expression by GCs. This allele may confer increased susceptibility to this autoimmune disease by allowing GCs to decrease *AIRE* expression.

In addition to these interacting polymorphisms, our analysis identified a much larger number of genes (6,813 genes) affected by no-interaction eQTLs (posterior probability>0.7; FDR = 0.16). In other words, transcript levels at these genes depend on the eQTL genotype, but the magnitude of transcriptional response does not (i.e. model 2, see Figure S10). Our observation that the vast majority of cis-acting regulatory polymorphisms with identical genotypic effects across treatment conditions is consistent with findings in osteoblasts treated with GCs [25] and in yeast [49], suggesting that this may reflect a general biological trend, rather than a feature specific to our treatment and experimental system. We compared the distribution of minor allele frequencies between the eQTLs following these three models and did not observe any significant differences (Figure S9).

The results reported above come from using a hierarchical model, which combines information across SNPs within each gene. One limitation of this hierarchical model is that it allows at most one eQTL per gene. This may cause it to miss interacting SNPs in genes that contain both interacting and non-interacting eQTLs, and for this reason the probabilities on interacting models may be underestimated. (More generally this feature could cause apparent discrepancies between the results from the hierarchical model and the log fold change analysis, although this does not seem to be the case in the results above.) To assess whether this limitation might have led us to miss some strong interaction signals we also performed a SNP-level analysis using the BF (for interaction models 2–4 vs non-interaction models 0–1) computed for each SNP. This analysis identified 247 SNPs, in 120 distinct genes, with BF exceeding 10^3^, although none exceeding 10^5^, that are candidates for being interacting eQTLs (Table S5).

### Validation of cis-regulatory polymorphisms with treatment-specific effects

To determine whether GC-only and control-only eQTLs represented regulatory polymorphisms with treatment-specific genotypic effects, we assayed treatment-dependent allelic imbalance using quantitative real time PCR in heterozygotes. This assay also asks whether local eQTLs act in cis, as alleles at cis-regulatory polymorphisms, by definition, affect target gene transcription only on the chromosome on which they reside. Among the 26 interaction eQTLs, we chose five at random among those for which a common coding SNP could be reliably genotyped. We assayed three genes with evidence of GC-only eQTLs (*C9orf5*, *LSG1*, and *MFGE8*). We found significant allelic imbalance, with allelic effects in the same direction as predicted by the eQTL mapping results, in GC-treated samples, but not in control-treated samples, for all of them (Table 1). We also performed allelic imbalance assays on 2 of the 8 control-only eQTLs (*SRD5A2*, *C12orf45*). We found significant allelic imbalance at *C12orf45* only in the control-treated samples. While not significant at p<0.05, we observed a pattern consistent with a control-only eQTL at *SRD5A2*. Our failure to fully validate all 5 assayed eQTLs by allelic imbalance could reflect some level of false positive identifications of eQTL interactions, but may also reflect incomplete power of the allelic imbalance assay.

**Table 1:** eQTL validation by allele-specific qRT–PCR.

Gene	eQTL Model	GC-treated		Control-treated	
		Effect	p-value	Effect	p-value
*C9orf5*	GC-only	0.12	**0.041**	−0.05	0.809
*LSG1*	GC-only	0.23	**8.38×10^−5^**	−0.01	0.585
*MFGE8*	GC-only	0.60	**0.033**	0.07	0.271
*C12orf45*	control-only	0.09	0.174	0.22	**0.017**
*SRD5A2*	control-only	0.01	0.445	0.95	0.118

Effects represent the natural log of the allelic ratio of qRT-PCR measurements of mRNA abundance, with the allele associated with increased expression in eQTL experiments as the numerator. P-values are from t-tests comparing allelic ratios between heterozygotes and homozygotes (as a control for technical sources of imbalance) at the eQTL. Significant p-values (p-value<0.05) are indicated in bold.

### eQTL replication in an independent study

We compared our results with those from an independent GC response eQTL mapping study in LCLs derived from asthma patients (W. Qui and K. Tantisira, personal communication). We found that 4 of the 9 interaction eQTLs that we identified, and that were tested in both studies, showed significant associations with log fold change in this independent dataset (p<0.05, *C1orf106*, *LSG1*, *CST7*, and *MS4A7*), and an additional 2 showed suggestive associations (p<0.1, *SYT17* and *BIRC3*). This overlap is highly significant (p = 8.5×10^−4^). Importantly, the overlap for single-treatment eQTLs is much greater than that for response eQTLs: all of the top 10 eQTLs identified by Qiu et al (2011) in each treatment condition were replicated in our data (p<0.05), while only 1 of the top 10 eQTLs for log fold change was replicated. This contrast highlights the known statistical challenge of mapping gene-environment interactions.

We also tested 15 of our interaction eQTLs (i.e. all eQTLs tested in both studies) for an association with response to GC therapy in 172 asthma patients (W. Qui and K. Tantisira, personal communication). We found that a GC-only eQTL for *TNIP1* was significantly associated with patient response (rs6870205, p = 2.5×10^−3^, Bonferroni-corrected p = 0.037). *TNIP1* has an established role in the immune response, as it encodes a protein that inhibits NFκB [50] and contains polymorphisms that have been associated with risk of systemic lupus erythematosus [46].

### Between-population variation in GC response

**Figure 3:**
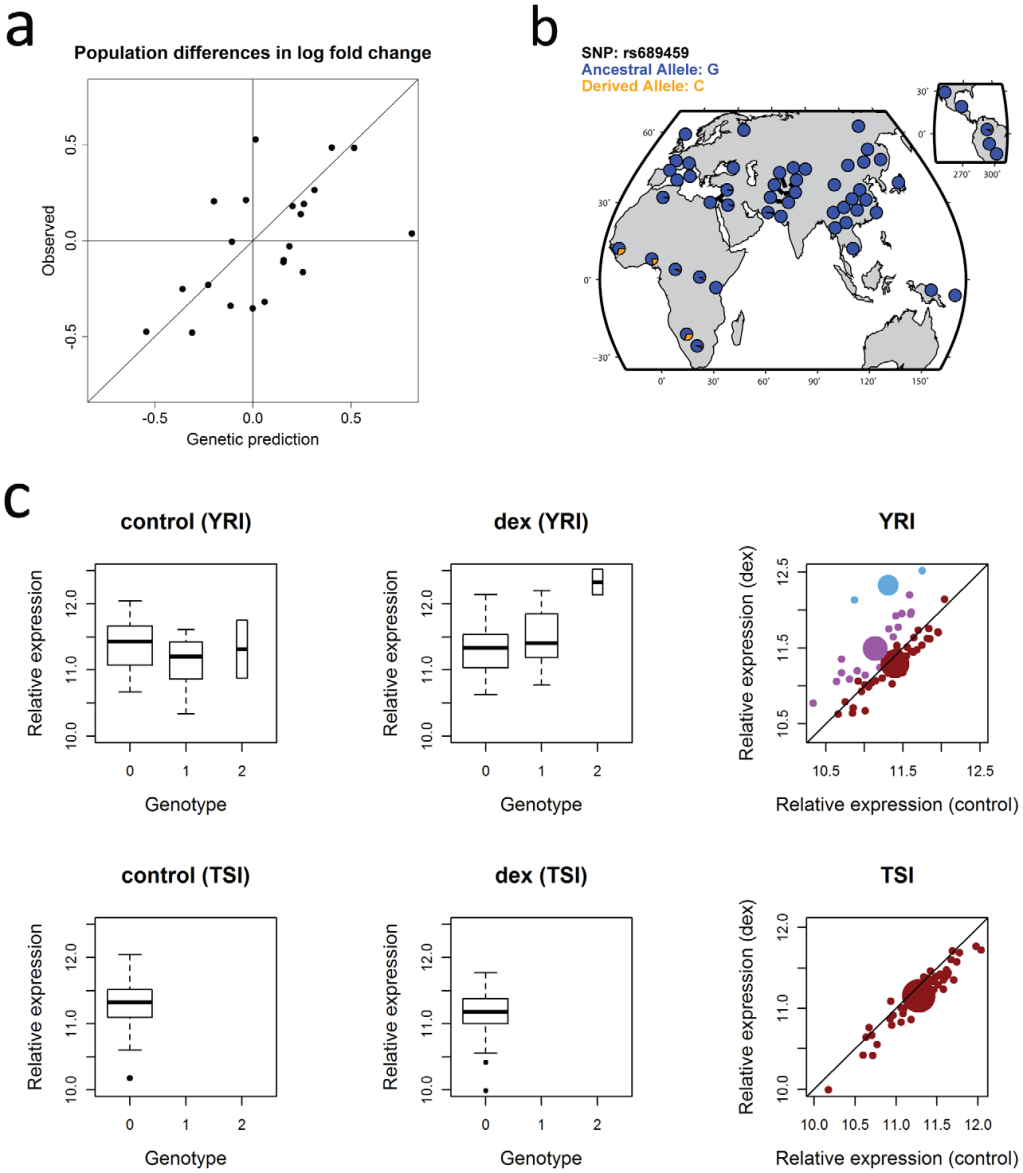
Population differences in transcriptional responses and allele frequency differences at an interaction eQTLs. a) Observed population differences in log-fold change (y-axis) are plotted against predictions based on genotypic effects and differences in allele frequency (x-axis). Genetic predicted values are significantly correlated with observed differences in response (r^2^ = 0.33, p = 5.3×10^−3^). b) The global distribution of the C allele at rs689459, which is associated with up-regulation of *NQO1* expression by GCs. c) The effect of the population-specific GC-only eQTL at *NQO1* by population. In plots on the far right, each small dot corresponds to an individual and is color-coded based on genotype (red = homozygous for G allele, purple = heterozygous, and blue = homozygous for C allele). Large dots represent genotypic means. Genotype is coded as copies of the C allele at rs689459. Relative expression corresponds to log_2_-transformed microarray intensities.

We observed substantial allele frequency differences between populations at many of the putative interaction expression quantitative trait nucleotides (eQTNs), defined as the most strongly associated SNP for each gene. Furthermore, differences in allele frequency at these eQTNs were predictive of differences in average transcriptional response between populations (r^2^ = 0.33, p = 5.3×10^−3^, Figure 3a). This demonstrates that these eQTNs contribute to differences in response between populations, and so may also contribute to inter-ethnic disparities in GC-related diseases and in drug response. It also provides independent supporting evidence that these eQTNs interact with GC treatment.

In some cases, allele frequency differences may explain why genes respond to GC treatment only in individuals of one population. For example, we observed that the GC-only eQTL allele associated with up-regulation of the detoxification enzyme NAD(P)H:quinone oxidoreductase 1 (*NQO1*) was extremely rare outside equatorial African populations (Figure 3b), likely causing the observed lack of *NQO1* response in TSI LCLs, and the strong up-regulation in many YRI LCLs (Figure 3c). This result may be of particular relevance to ethnic disparities in leukemia patient response to GCs, as alleles that reduce *NQO1* enzymatic activity have been associated with decreased response to a chemotherapy regime that included GCs in patients with acute lymphoblastic [51], [52] and acute myeloid leukemia [53].

In an effort to identify additional genes with differences in average transcriptional response between populations, we applied the same statistical framework described above to test for interactions between population (rather than genotype) and GC treatment. Using this approach, we identified 258 genes with differences in transcriptional response (posterior>0.7, FDR = 0.128) between populations; of these, 130 were up-regulated by GC treatment while 128 were down-regulated. We found a consistent pattern across genes, with a tendency for stronger up-regulation in YRI LCLs at 78% of up-regulated genes with population differences in response (Figure S3). Interacting eQTLs are enriched among genes with population differences in response compared to all expressed genes (odds ratio = 6.0, p = 5.4×10^−3^) while no-interaction eQTLs are not enriched (odds ratio = 0.99). 

### IL6 secretion is affected by a GC-only cis-regulatory polymorphism

**Figure 4:**
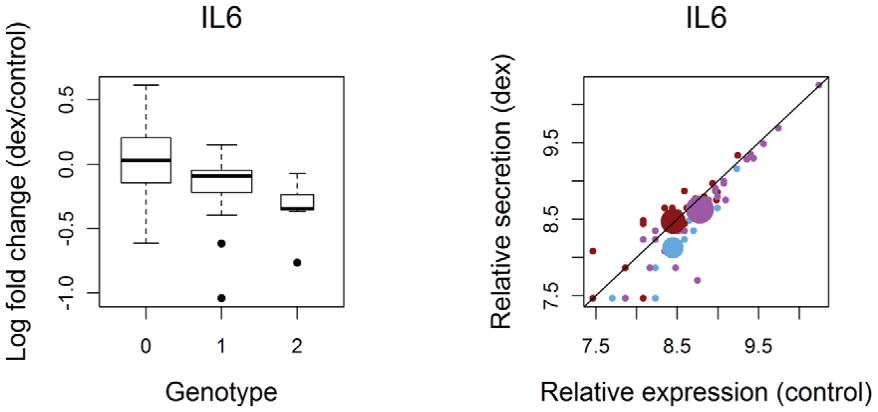
Secretion QTL for IL6. Genotype is coded as copies of the minor allele. Log fold change in secretion corresponds to the difference between dex and control of covariate-corrected, quantile-normalized, log_2_-transformed estimates of relative quantity from ELISA assays. In plot on the right, each small dot corresponds to an individual and is color-coded based on genotype (red = homozygous for major allele, purple = heterozygous, and blue = homozygous for minor allele). Large dots represent genotypic means.

The attenuation of the immune response by GCs is partially mediated by decreased secretion of pro-inflammatory molecules. We measured the secreted levels of 9 pro-inflammatory proteins (IL1α, IL6, IL8, IP10, MDC, Rantes, TNFα, TNFβ) and 1 anti-inflammatory protein (IL10). Five pro-inflammatory proteins showed significant differential secretion in response to GCs in LCLs (TNFα, TNFβ, Rantes, IP10 and IL1α –Table S3); all five showed lower secretion levels in the presence of GC, consistent with the immune-repressive role of GCs. To identify genetic variation that influences GC-mediated regulation of protein secretion, we tested HapMap SNPs for association with log fold change in secretion at each protein. Similar to our eQTL results, we found significant associations (at a FDR<0.2) only when we limited our search to SNPs near the genes that encode each protein (i.e. we found no significant associations in genome-wide or a candidate gene analysis). Testing SNPs within 100 kb of each cytokine, we found a significant association between secretion response at IL6 and genotype at a SNP ∼56 kb downstream (rs10225286, p = 1.9×10^−4^, FDR = 0.1, Figure 4). Because this SNP did not show strong evidence of an effect on IL6 transcriptional response, we propose that it affects secretion through a mechanism independent of mRNA levels or that it affects transcriptional response at a different treatment time point.

## Discussion

Here, we report a genome-wide scan for genetic variation that influences the GC-mediated regulation of transcription and protein secretion. The cellular response to GCs depends on a well-characterized set of regulatory proteins (i.e. the GR and interacting proteins). This provided us with a set of strong candidate loci to perform trans-eQTL mapping tests. Despite this, we found no evidence for trans-acting factors. In contrast, the strongest signal from an unbiased genome-wide scan was a SNP associated with transcriptional response at a nearby gene, and even more eQTLs were revealed when we limited our analysis to SNPs within 100 kb of each gene. Numerous studies have tested genetic variation within or near the GR and interacting transcription factors for association with patient response to GC treatment. These studies have found mostly rare functional polymorphisms that are unlikely to explain most heritable variation in GC response (reviewed in [54]). Furthermore, rare polymorphisms in the GR have dramatic phenotypic effects (e.g. extreme hypoglycemia and hypertension [55]), as expected for a master regulator that influences all downstream processes. Instead of genetic variants in master regulators, our results suggest that cis-regulatory polymorphisms that interact with GC treatment at target genes could play an important role in GC response, as first suggested based on observations at the *SGK1* gene [56]. These findings suggest that future attempts to identify genetic variation associated with clinical response to GCs may benefit from focusing on likely cis-regulatory polymorphisms that impact response at individual GC target genes, instead of testing master regulators of the GC response pathway.

We found that associations between genotype and transcriptional response could be discriminated into distinct categories based on the configuration of genotypic effects across treatment conditions. These categories likely correspond to specific genetic mechanisms. GC-only eQTLs may reflect polymorphisms that influence the binding of transcription factors that are only active in the presence of GC treatment (e.g. the GR and interacting transcription factors). In support of this hypothesis, we found that GC-only eQTLs tended to affect up-regulated genes (13 of 18). Although the causative polymorphism may not be among the genotyped SNPs, we found examples of GC-only eQTLs where most of the signal centered on a SNP that disrupts a predicted GR binding site, such as the eQTN for *C9orf5* (rs10816772, p for motif match = 6.8×10^−3^).

Control-only eQTLs are compatible with a variety of mechanisms. For example, they may reflect polymorphisms that disrupt the binding of regulatory complexes, like NFκB, that are directly inhibited by the GR (e.g. through protein-protein interaction). Consistent with this, we found examples of control-only eQTLs where most of the signal centered on a SNP that disrupts a predicted binding site for a transcription factor directly inhibited by GR, such as the eQTN for *FBXL6* (rs10448143, matrix and core similarity for NFkB>0.9). Direct inhibition of transcription factors by GR generally leads to down-regulation of target genes. However, we found equal numbers of control-only eQTLs affecting up-regulated and down-regulated genes (4 of each), so additional mechanisms must explain some fraction of control-only eQTLs. These may include genetic effects on regulatory elements that are indirectly inhibited by GC treatment (e.g. through GR competition for access to DNA by another transcription factor) or polymorphisms that affect transcriptional response at secondary targets.

The different categories of interactions identified by our method may also have distinct phenotypic interpretations. Polymorphisms with GC-only effects on expression are likely to directly affect the action of the GC-activated regulatory machinery. In contrast, polymorphisms with control-only effects have no impact on the cellular processes in the presence of GCs, but may still affect phenotype by influencing variation in a ‘pre-treatment’ state. For example, genetic effects on pro-inflammatory cytokine levels prior to GC exposure could affect the amount of time cells take to reach the optimal, lower levels required to effectively suppress inflammation. In summary, control-only QTLs may contribute more to variation in underlying disease mechanisms, while GC-only QTLs may contribute to variation in GC pharmacodynamics. However, we also note that, given their lower rates of validation and replication, there may be a higher false positive rate for control-only eQTLs.

Inter-ethnic differences in GC response have been observed clinically [13], [14], and the prevalence of many GC-regulated physiological traits differs across human populations [57]. By combining association mapping with comparisons between populations, our study allowed a direct assessment of the genetic basis of population differences in the cellular response to GCs. We found that ancestry had substantial and systematic effects on the transcriptional response to GCs, with a tendency for stronger up-regulation after GC treatment in YRI LCLs. Possible causes of such patterns include: non-genetic ‘confounders’ (e.g. differences in immortalization procedure [58]), trans-acting alleles that increase response and are at higher frequency in YRI, or multiple, independent cis-acting alleles that increase response in YRI at up-regulated genes. Our data favor the last explanation. It seems unlikely that non-genetic ‘confounders’ explain all or most of the population differences, as we found that the measured ‘confounders’ showed limited evidence of effects on transcriptional response or differences between populations (Figure S5). Although we cannot exclude the possibility that population differences reflect a trans-acting eQTL with differences in allele frequency, we found little support for this explanation. Instead, we found evidence suggesting that population differences may reflect differences in allele frequency at cis-regulatory polymorphisms, as genes with population differences in response were more likely to have local interaction eQTLs. The possibility that a stronger response in YRI reflects differences in allele frequency at cis-regulatory polymorphisms is particularly interesting from an evolutionary perspective, as differences in allele frequency acting in a consistent direction (i.e. increasing GC responsiveness) across multiple independent QTLs are usually interpreted as evidence of polygenic adaptation [59]–[61].

In addition to these biological insights, we contribute novel statistical methodology for mapping response phenotypes and identifying gene-environment interactions. These methods are applicable for any setting contrasting genotypic effects between two conditions (with paired measurements), including pharmacogenetic studies of clinical response to drug therapy (e.g. [62]) and, especially, functional genomic studies of genetic effects on treatment response similar to the one presented here. These methods provide a more powerful alternative to mapping a measure of response (e.g. log fold change), which fails to distinguish among different types of interactions, or comparing results from mapping separately in each condition, which ignores the paired nature of the data.

In summary, this study provides an initial characterization of the genetic basis of variation within and between human populations for a key physiological regulator and commonly administered pharmaceutical. The biological insights and statistical tools presented here extend our current understanding of the genetic basis of variation in response to GCs, and will aid future efforts to characterize the genetics of response to this and other treatments.

## Materials and Methods

### Cell culture and dexamethasone treatment

All cellular experiments described were conducted in lymphoblastoid cell lines (LCLs), B lymphocytes transformed with Epstein-Barr virus, that were collected as a part of the International HapMap project. LCLs were thawed and passed once in RPMI media supplemented with 15% fetal bovine serum, then washed twice with phosphate-buffered saline and moved to RPMI media supplemented with 15% charcoal-stripped fetal bovine serum. After one passage in media with charcoal-stripped fetal bovine serum (corresponding to a minimum culturing time of 5 days), LCLs were seeded in the evening at a density of 5×10^5^ cells/ml. After an overnight incubation, LCLs were treated with 10^−6^ M dexamethasone, and an equal amount of vehicle solution (solution composed of 1% ethanol and 99% cell culture media) as a negative control for treatment. For each LCL, one set of dex and control aliquots was treated for 8 hours (to quantify mRNA abundance) and the other for 24 hours (to assay inflammatory protein secretion). The study design is depicted in Figure S6. LCLs were thawed, cultured and treated in batches completely balanced by treatment, population, technician and time of day. For quality control purposes, biological replicates were performed for one batch of four cell lines and both expression and treatment response were highly replicable (Figure S7). Collection of all samples took 4 months.

### RNA extraction and array hybridization

For each expression study described in the preliminary data, total RNA was extracted from each cell culture sample using the QIAgen RNeasy Plus mini kit, and was found to be of high quality. RNA was extracted from all 240 samples over the course of 5 days. Total RNA was then reverse transcribed into cDNA, labeled, hybridized to Illumina HumanHT-12 v3 Expression BeadChips and scanned at the Southern California Genotyping Consortium (SCGC: http://scgc.genetics.ucla.edu/) at the University of California at Los Angeles. Each RNA sample was hybridized to two separate arrays (i.e. in two technical replicates). To avoid batch effects on RNA measurements, all 480 microarrays were hybridized within 7 days. Summary data (e.g. mean intensity of each probe across within-array replicates) were obtained using the BeadStudio software (Illumina) at the SCGC. The microarray data has been deposited in the Gene Expression Omnibus (GEO), www.ncbi.nlm.nih.gov/geo, under accession number GSE29342.

### Low-level analysis of microarray data

Low-level microarray analysis was performed using the Bioconductor software package LUMI [63] in R (http://www.r-project.org). We used applied variance stabilizing transformation [64] to all arrays, removed probes with intensities indistinguishable from background fluorescence levels in all samples (leaving 23,700 expressed probes), and performed quantile normalization across all arrays. Probes were annotated by mapping to the RNA sequences from RefSeq using BLAT. To avoid ambiguity in the source of a signal due to cross-hybridization of similar RNA species, probes that mapped to multiple genes were excluded from further analyses. Probes that contained one or more HapMap SNPs were also removed from further analyses to avoid spurious associations between expression measurements and SNPs in linkage disequilibrium.

### Measurement and correction for confounders

To avoid spurious results and to reduce noise due to potential confounders, we measured several covariates relevant to LCL biology including: EBV genome copy number, growth rate and mitochondrial genome copy number. EBV and mitochondrial genome copy number were assessed using Taqman Gene Expression Assays (Assay # Hs02596867_s1 for mitochondria and Pa03453399_s1 for EBV). RNaseP was used as an endogenous control for both assays. We then used linear regression to remove the effects of these potential confounders at each gene and confounder-corrected data were used in all subsequent analyses.

### Identification of differentially expressed genes

In order to identify genes that, on average across individuals, changed expression levels upon treatment with GCs, we performed multiple linear regression at each gene with treatment as the covariate of interest while taking other measured covariates into account. To reduce the effects of outliers, microarray intensity values were quantile normalized to a *N(0,1)* distribution across all samples (treated and untreated). We used the distribution of p-values observed when sample labels are permuted (ten permutations were used), an empirical estimate of the p-value distribution under the null, to estimate the false discovery rate (FDR). We used the online tool DAVID [65], [66] to identify biological categories enriched among differentially expressed genes, using all genes expressed in LCLs (based on microarray data) as a background.

### SNP imputation

We used all HapMap SNPs for all mapping experiments described. As TSI LCLs were only typed for phase III SNPs, we used the CEU population sample to impute genotypes at all HapMap phase I and II SNPs. Similarly, we imputed SNPs for phase III YRI LCLs based on the YRI LCLs included in phase I and II. Imputation was performed using BIMBAM [67], which infers missing genotypes based on correlations between missing and typed genotypes observed in samples where all genotypes are typed. QTL mapping results were not qualitatively different if using imputed or genotyped SNPs.

### Genetic mapping of log-fold change in expression

We tested for association between all HapMap SNPs and transcriptional response at each gene, using log fold change in expression (GC-treated over control-treated expression) as a measure of response. For our candidate gene-based scan for trans-acting eQTLs that influenced response, we tested all HapMap SNPs within 500 kb and 100 kb (in two separate sets of analyses) of genes encoding the GR and transcription factors that interact with the GR. Interacting transcription factors include the genes that encode the components of the NFkB complex, AP1, Oct1, Oct2, CREB, ETS1, STAT3, STAT5, STAT6, C/EBP, TFIID, T-bet, PU.1/Spi-1, Smad3, Smad4, Smad6, COUP-TFII, IRF3, STIP1, Hic5/Ara55, and nTrip6 [29]. P-values calculated with permutated genotype labels were used as an empirical null distribution. In order to maintain the correlation structure across genes, the same permutation seed was used for all genes in both candidate gene tests and the genome-wide scan. Ten permutations were performed for the test of variation within 500 kb, 100 permutations were used for the test of variation within 100 kb and 3 permutations were used for the genome-wide scan. For mapping log fold change at SNPs within 500 kb or 100 kb of each gene, permutation seeds were set separately at each gene. Association tests were performed using a combination of Python, the R statistical package and the genetic association mapping program PLINK.

### Bayesian regression for identifying genetic associations and interaction with treatment

We developed a novel Bayesian statistical framework for genetic association analysis in settings where measurements are available on the same individuals in two different conditions (in our case, GC-treated and control-treated). Our methods extend and improve the methods from Barber et al. (2009) to explicitly consider “qualitative interaction” models where genetic variants are associated with measurements in only one of the two conditions. Our method takes into account both sample pairing and the intra-individual correlation of measurements under the two conditions. We describe our method in greater detail in Text S1. These methods are implemented in software called BRIdGE (Bayesian Regression for Identifying Gene-Environment interactions), which is available on the Stephens and the Di Rienzo laboratories' web pages (http://stephenslab.uchicago.edu/software.html, http://genapps.uchicago.edu/labweb/index.html).

### Allele-specific quantitative PCR on cDNA to assay allelic imbalance

We used TaqMan quantitative genotyping assays to test for allelic imbalance at coding SNPs in LD with eQTLs that interacted with GC treatment. Imbalanced expression of the two coding alleles is an independent line of evidence for a cis-acting regulatory polymorphism and for the configuration of the effect in the two treatment conditions (i.e. the interaction model). Total RNA from an aliquot of the same culture samples used to hybridize microarrays (this was a separate RNA extraction as that used to hybridize microarrays) was synthesized into cDNA using the High-Capacity cDNA Reverse Transcription Kit (Applied Biosystems, Foster City, CA) according to the manufacturer's protocol. Taqman SNP Genotyping Assays were used to quantify relative mRNA abundance of each allele on an ABI PRISM 7900HT Sequence Detection System. To account for differences between the two fluorochromes, a standard curve was built for each of the two alleles using serial dilutions of a genomic DNA from an individual that was heterozygous at the coding SNP. For each assay, we calculated the natural log-ratio between the two different alleles. The numerator of this ratio was always the allele associated with increased expression in the corresponding treatment condition. Within each treatment, we quantile normalized allelic log-ratios and used a one-tailed t-test to identify significant differences in average allelic log-ratios between heterozygotes and homozygotes (as an empirical null distribution of allelic log-ratios) at the eQTL.

### Overlap with other genetic association studies

We compared our eQTL results to multiple genetic association studies including Qiu et al. (2011) and those in the GWAS catalog. For each interaction eQTL, we compared evidence at the most associated SNP in our data when it was tested in both studies. When the most associated SNP was not tested in the comparison dataset, we identified the best proxy SNP for each eQTL among those tested in both studies. To ensure that the best proxy SNP captured the pattern at the original eQTL, we required the proxy SNP to show strong evidence of association for the same eQTL model as the original eQTL (BF for association>500 and posterior probability for model>0.5).

### Comparing transcriptional response between populations

We contrasted the transcriptional response to GCs between YRI and TSI LCLs. Differences in transcriptional response between populations will result in differences in average expression levels that differ depending on treatment, as opposed to GC-independent population differences that will be identical in both treatments. As this is analogous to gene-environment interactions, we used the same statistical framework to identify genes with differences in transcriptional response between populations (see Bayesian regression for identifying genetic associations and interaction with treatment in Text S1). Covariate-corrected expression levels were quantile normalized across individuals (both YRI and TSI) for each gene to reduce the effect of outliers. As population differences at the phenotypic level may reflect population differences in response following a consistent pattern across many genes, we identified the direction of population differences at each gene in terms of log-fold change.

### Quantification of inflammatory markers in the cell culture medium and identification of secretion QTLs that interact with GC treatment

A multianalyte ELISA assay (Millipore) was performed on the culture medium of the cell aliquots treated for 24 hours. The assay was performed at the Flow Cytometry Facility at the University of Chicago, according to the manufacturer instructions. Two technical replicates were run for each sample. Samples were assayed in batches balanced by treatment and population. For each analyte, the average quantity across technical replicates was calculated and used for all subsequent analyses. The correlation structure between paired aliquots for each sample (GC and control) was visually inspected (Figure S8). A small subset of samples with low quantity detected showed no correlation between GC and control aliquots because of noise in the measurement at low concentrations. Consequently, these samples were excluded from downstream analyses. Secretion levels were highly correlated across proteins, likely representing a latent factor that generally affects secretion levels. To remove the effect of this latent factor, we used linear regression to correct secretion levels at each protein by secretion levels at all other measured proteins.

## Supporting Information

Figure S1 Transcriptional response to GC treatment in LCLs. a) We identified 4,568 differentially expressed genes including up- and down- regulated genes. b) Sub-sampling shows that the number of differentially expressed genes identified is a function of sample size. Larger sample sizes tend to identify genes with c) more variable and d) smaller responses. Aberrantly high, relative to the overall trend, median coefficients of variation and low log-fold changes at the smallest sample size (n = 4) likely reflect increased sampling noise due to a very small sample size.
(TIF)

Figure S2 Results from Bayesian method for mapping interactions with treatment is compared to the traditional frequentist test for association with response (i.e. association between genotype and log-fold change). The quantiles of the observed p-value distribution (minimum p-value per gene) are plotted against expected quantiles (based on 10 permutations). The p-value threshold corresponding to a FDR<0.1 is marked by a horizontal grey line. Genes with significant interaction eQTLs identified by the Bayesian multivariate method at a posterior>0.7 are shown in red for the GC-only model and in blue for the control-only model. Interactions identified by our method include both top hits from the frequentist analysis and a number of additional genes, indicating that our approach provides increased in power to detect polymorphisms that interact with treatment and affect response.
(TIF)

Figure S3 A stronger response to GCs is observed in YRI LCLs at up-regulated genes. Strength of response, measured by log-fold changes (GC/control), is depicted by intensity and color, with red corresponding to lower log-fold change and blue corresponding to higher log-fold change, for a) up-regulated and b) down-regulated genes with significant population differences in transcriptional response. Rows represent genes and columns represent individuals. The vertical black lines represent the separation between the populations.
(TIF)

Figure S4 Observed p-values for association between genotype and log-fold change in expression (GC/control) are plotted against p-values from permutations, representing expectations under the null, for a) SNPs within 500 kb (only SNP with minimum p-value is plotted for each gene) of the gene that encodes the GR and b) SNPs within 500 kb of transcription factors known to interact with the GR. c) SNPs within 500 kb of each of the interacting transcription factors and d) within 100 kb of each of the interacting transcription factors.
(PDF)

Figure S5 Differences between TSI and YRI LCLs in the distribution of factors known to affect LCL biology. We do not observe significant differences in a) EBV genome copy number (p = 0.824) or b) growth rate (p = 0.477). c) We did observe a significantly higher level of mitochondrial genome copy number among YRI LCLs (p = 0.0463).
(TIF)

Figure S6 The study design used in this experiment is shown. For each of 116 LCLs, one aliquot was treated with the synthetic GC dexamethasone and another aliquot was treated with the vehicle for dexamethasone (EtOH) as a treatment control. Two sets of paired aliquots were treated for each LCL, one for 8 hours and the other for 24 hours. RNA was extracted from aliquots treated for 8 hours and hybridized to two replicate arrays (for a total of 4 arrays hybridized per LCL). Supernatant from the aliquots treated for 24 hours were used to assay protein secretion.
(TIF)

Figure S7 a) Pair-wise correlations between technical replicates (red), representing duplicate RNA hybridizations, tend to be larger than correlations between randomly drawn pairs of arrays (grey), suggesting a limited contribution from RNA hybridization to variation in these measurements. b) Pair-wise correlations of expression levels between biological replicates are always larger (red) than randomly drawn pairs of LCLs (grey), similarly suggesting that variation in cell culturing and treatment protocols described here contribute little to variation in expression measurements. c-d) Log-fold changes (GC/control) across the 4,568 differentially expressed genes compared between biological replicates to assess the reproducibility of response. c) Correlations between biological replicates (red) are higher than expected when comparing randomly drawn pairs of LCLs (grey), suggesting that variation from cell culturing and treatment does not explain the majority of variation in response between LCLs. d) Correlations between replicate pairs are shown for transcriptional response across differentially expressed genes.
(TIFF)

Figure S8 Correlation between secretion levels across individuals for each protein. Secretion levels represent log-transformed ELISA measurements of protein quantity. Horizontal and vertical lines in each plot indicate the threshold used to identify meaningful secretion measurements.
(TIF)

Figure S9 Distribution of minor allele frequency for each candidate eQTN categorized by model. No significant difference is observed, based on Mann-Whitney U test, between control-only and GC-only eQTNs (p = 0.26), GC-only and no-interaction eQTNs (p = 0.94), or control-only and no-interaction eQTNs (p = 0.19).
(TIF)

Figure S10 Quantile-quantile plots showing the distribution of p-values from mapping the log fold change and log sum (sum of log expression values in GC-treated and control-treated samples) at genes affected by no-interaction eQTLs. No deviation from null expectations are observed for association with log fold change, while a very strong deviation from expectations under the null is observed for association with the log sum. This is consistent with the stable effect of eQTL genotype on expression at these genes. SNPs within 100 kb were tested against log fold change at each gene. Observed minimum p-values per gene are shown as black dots. Minimum p-values from permutations are shown as grey dots.
(TIF)

Table S1 Association between gene expression and EBV copy number, mitochondrial copy number, and growth rate.
(PDF)

Table S2 eQTLs that interact with GC treatment.
(PDF)

Table S3 Effects of GC treatment on cytokine secretion.
(PDF)

Table S4 Maximum likelihood estimates of the proportion of genes with an association following each model for all the predictor variables we tested against gene expression.
(PDF)

Table S5 SNPs showing strongest signal for interaction from the SNP-based Bayesian analysis.
(PDF)

Text S1 Supplementary Materials and Methods.
(PDF)
