# Supplementary figures and images for: Interactions between Glucocorticoid Treatment and Cis-Regulatory Polymorphisms Contribute to Cellular Response Phenotypes
Source: PLoS Genet. 2011 Jul 7;7(7):e1002162. doi: 10.1371/journal.pgen.1002162 (PMC3131293; doi:10.1371/journal.pgen.1002162)

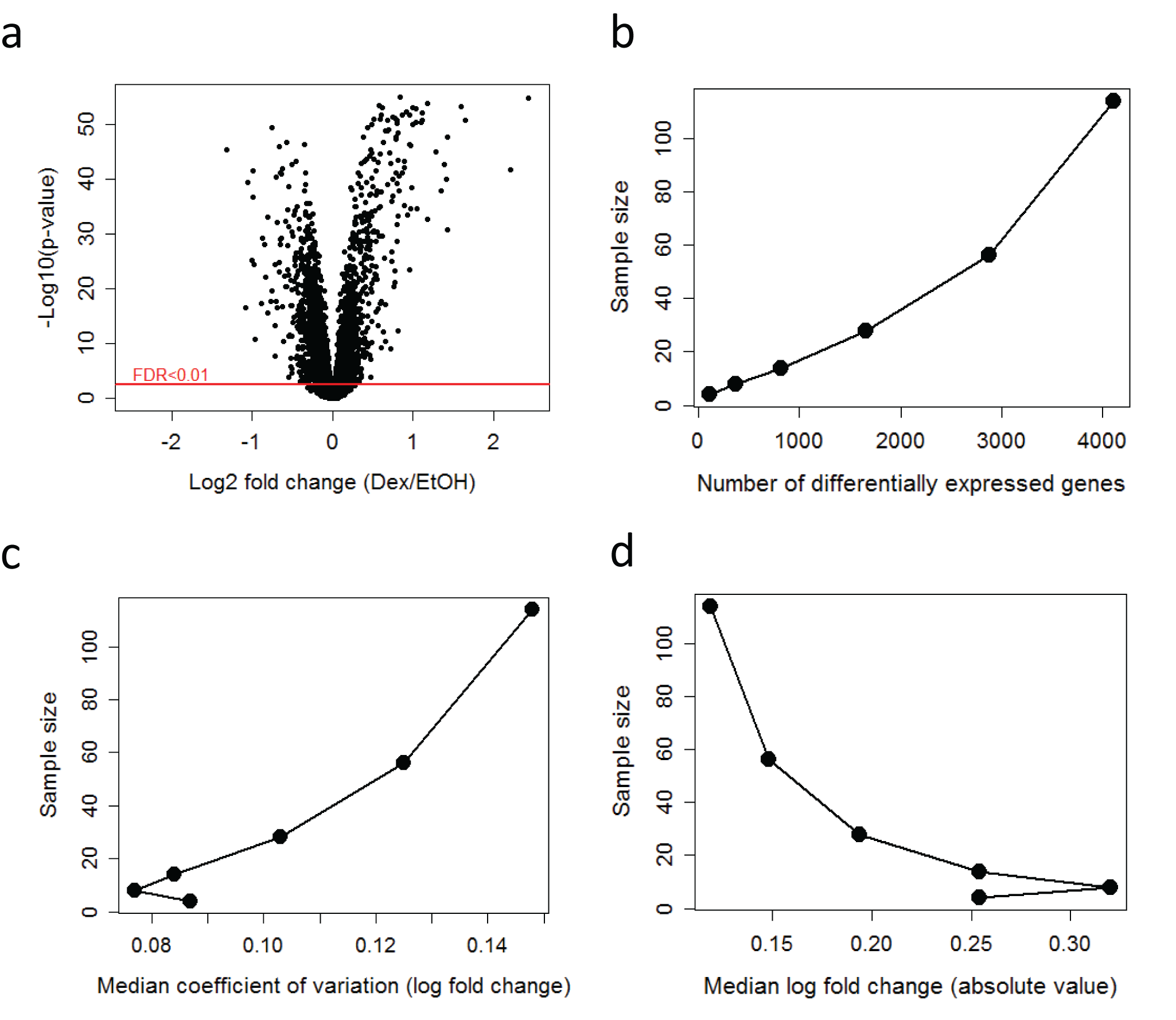

Supplement: Figure S1 — Transcriptional response to GC treatment in LCLs. a) We identified 4,568 differentially expressed genes including up- and down- regulated genes. b) Sub-sampling shows that the number of differentially expressed genes identified is a function of sample size. Larger sample sizes tend to identify genes with c) more variable and d) smaller responses. Aberrantly high, relative to the overall trend, median coefficients of variation and low log-fold changes at the smallest sample size (n = 4) likely reflect increased sampling noise due to a very small sample size. (TIF) [file pgen.1002162.s001.tif]

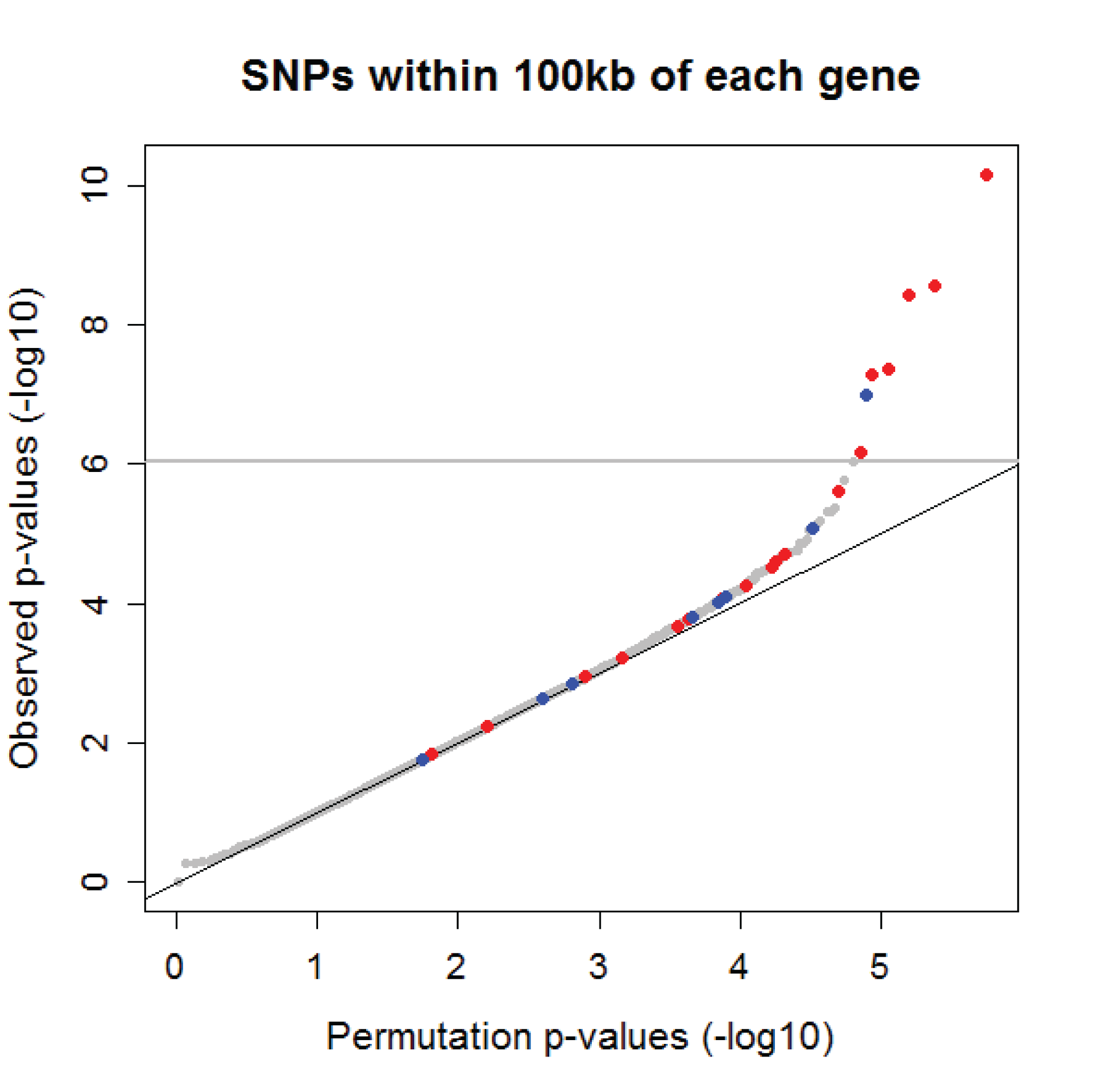

Supplement: Figure S2 — Results from Bayesian method for mapping interactions with treatment is compared to the traditional frequentist test for association with response (i.e. association between genotype and log-fold change). The quantiles of the observed p-value distribution (minimum p-value per gene) are plotted against expected quantiles (based on 10 permutations). The p-value threshold corresponding to a FDR<0.1 is marked by a horizontal grey line. Genes with significant interaction eQTLs identified by the Bayesian multivariate method at a posterior>0.7 are shown in red for the GC-only model and in blue for the control-only model. Interactions identified by our method include both top hits from the frequentist analysis and a number of additional genes, indicating that our approach provides increased in power to detect polymorphisms that interact with treatment and affect response. (TIF) [file pgen.1002162.s002.tif]

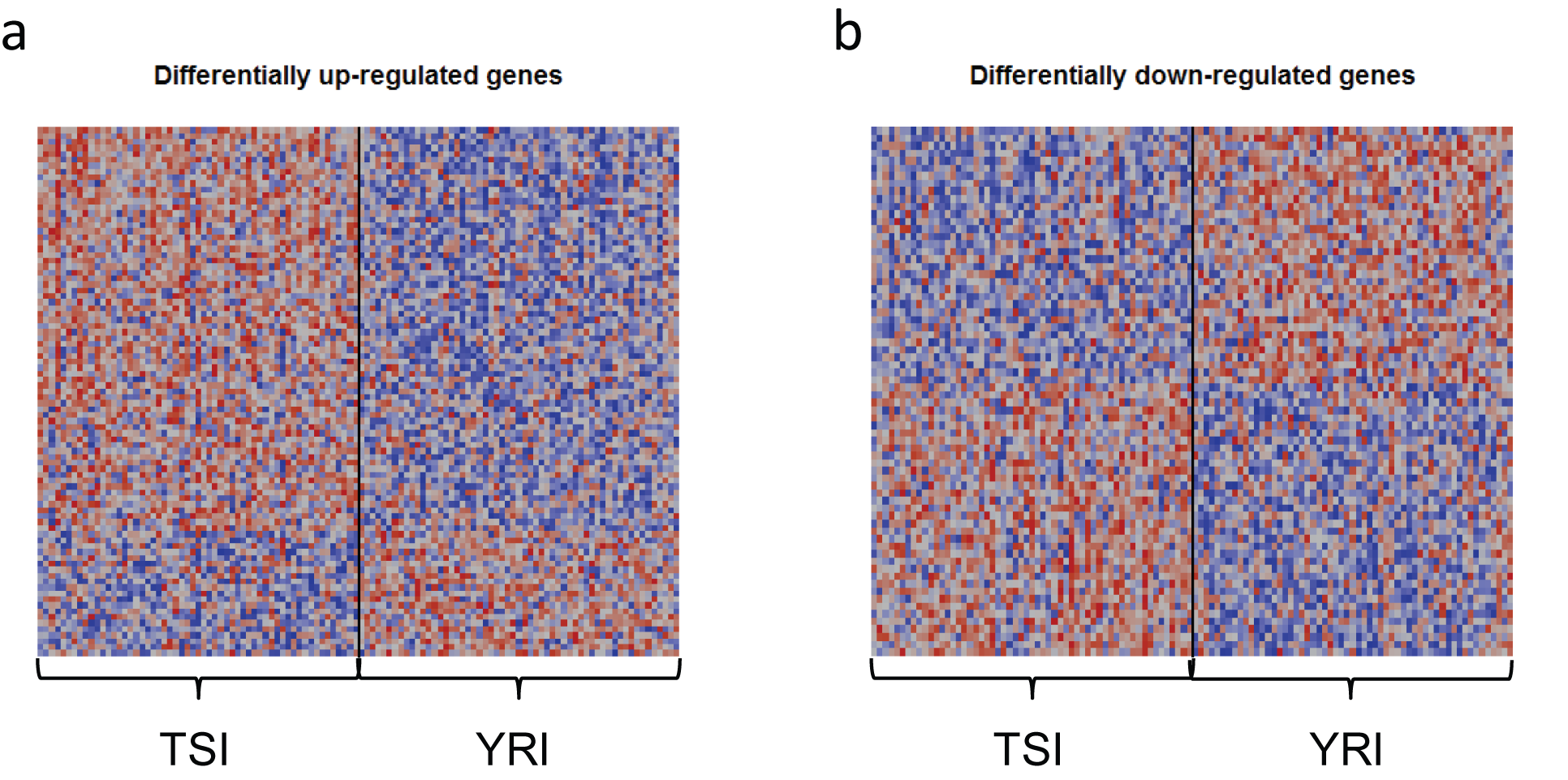

Supplement: Figure S3 — A stronger response to GCs is observed in YRI LCLs at up-regulated genes. Strength of response, measured by log-fold changes (GC/control), is depicted by intensity and color, with red corresponding to lower log-fold change and blue corresponding to higher log-fold change, for a) up-regulated and b) down-regulated genes with significant population differences in transcriptional response. Rows represent genes and columns represent individuals. The vertical black lines represent the separation between the populations. (TIF) [file pgen.1002162.s003.tif]

**a****SNPs within 500kb of GR**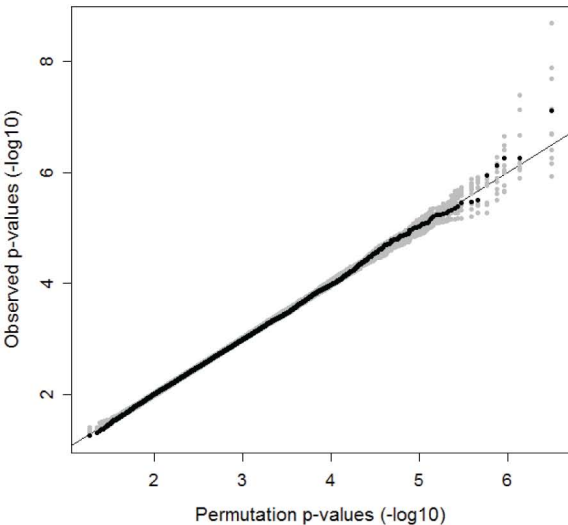**b****SNPs within 500kb of TFs that interact with GR**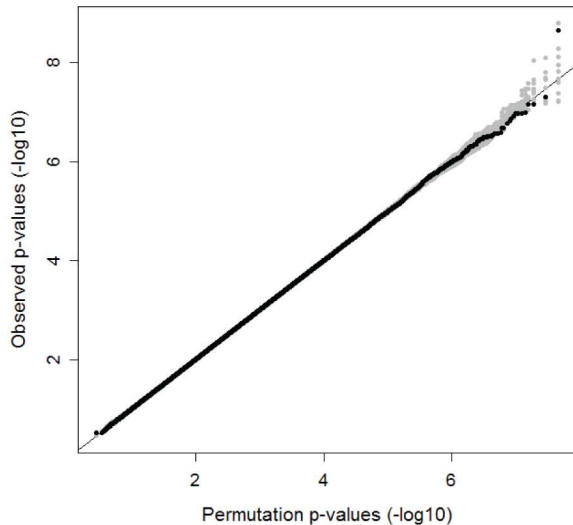

C

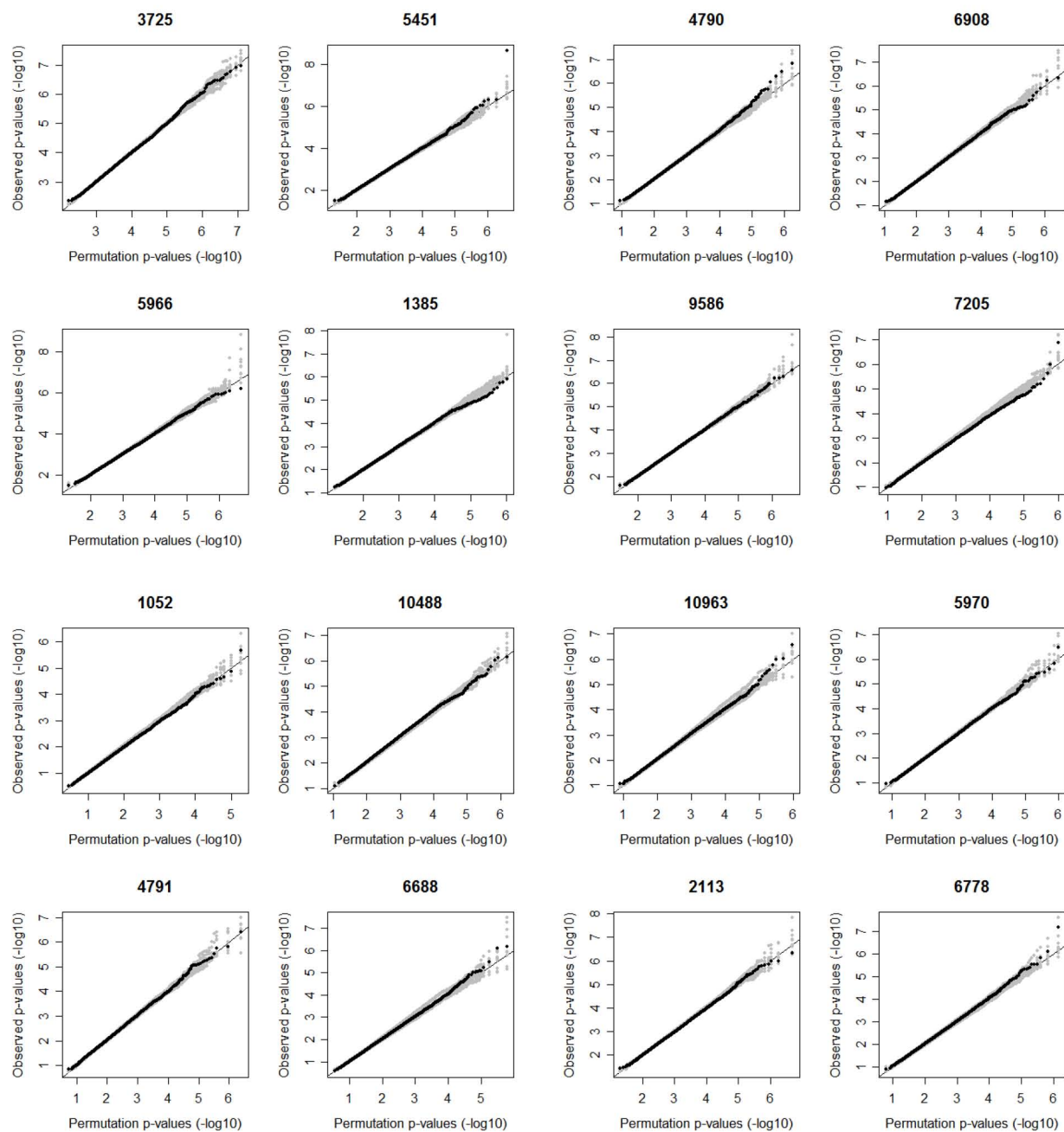

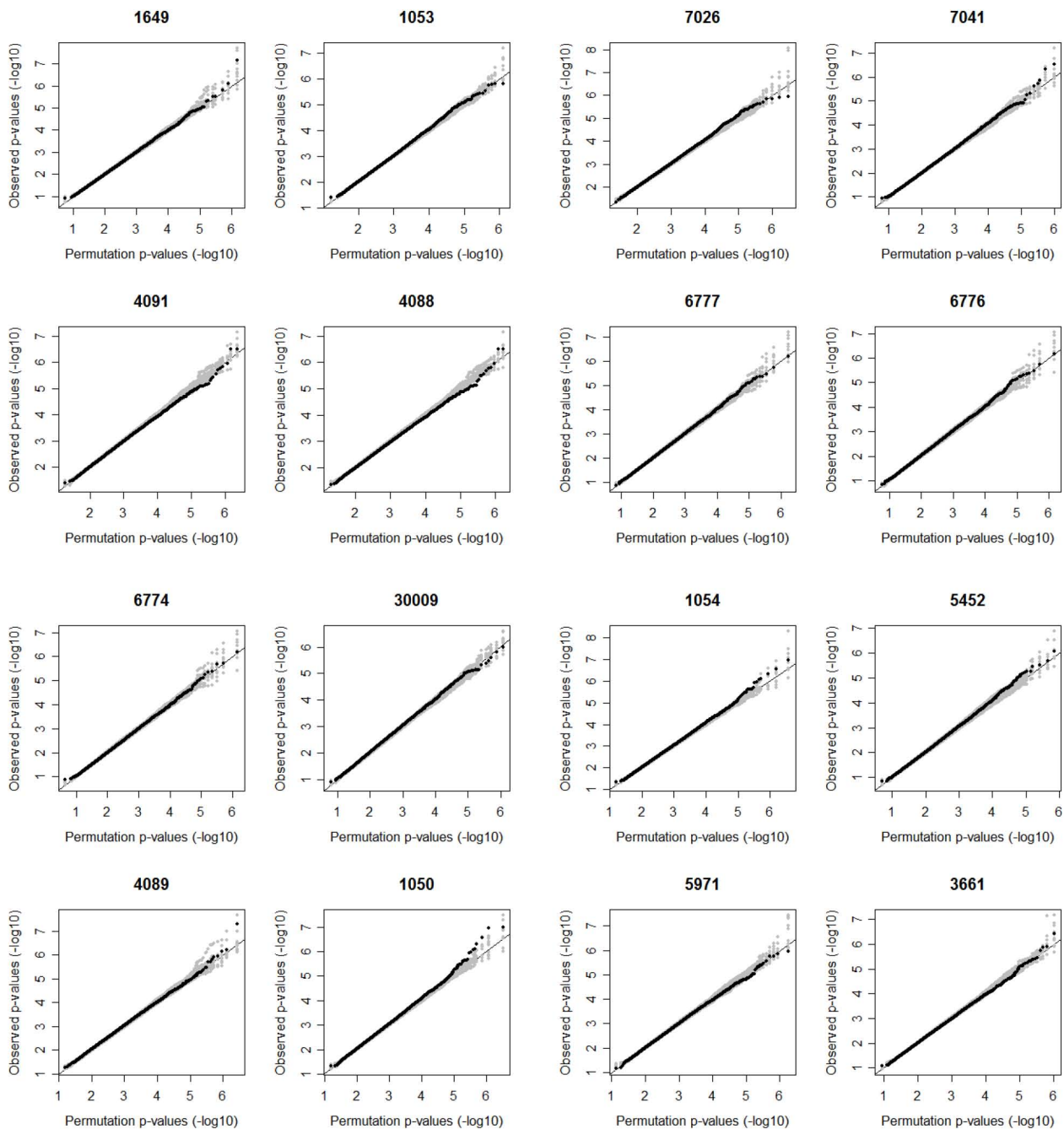

1051

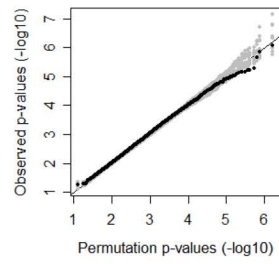

468

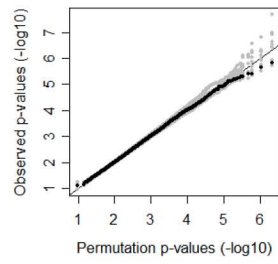

d

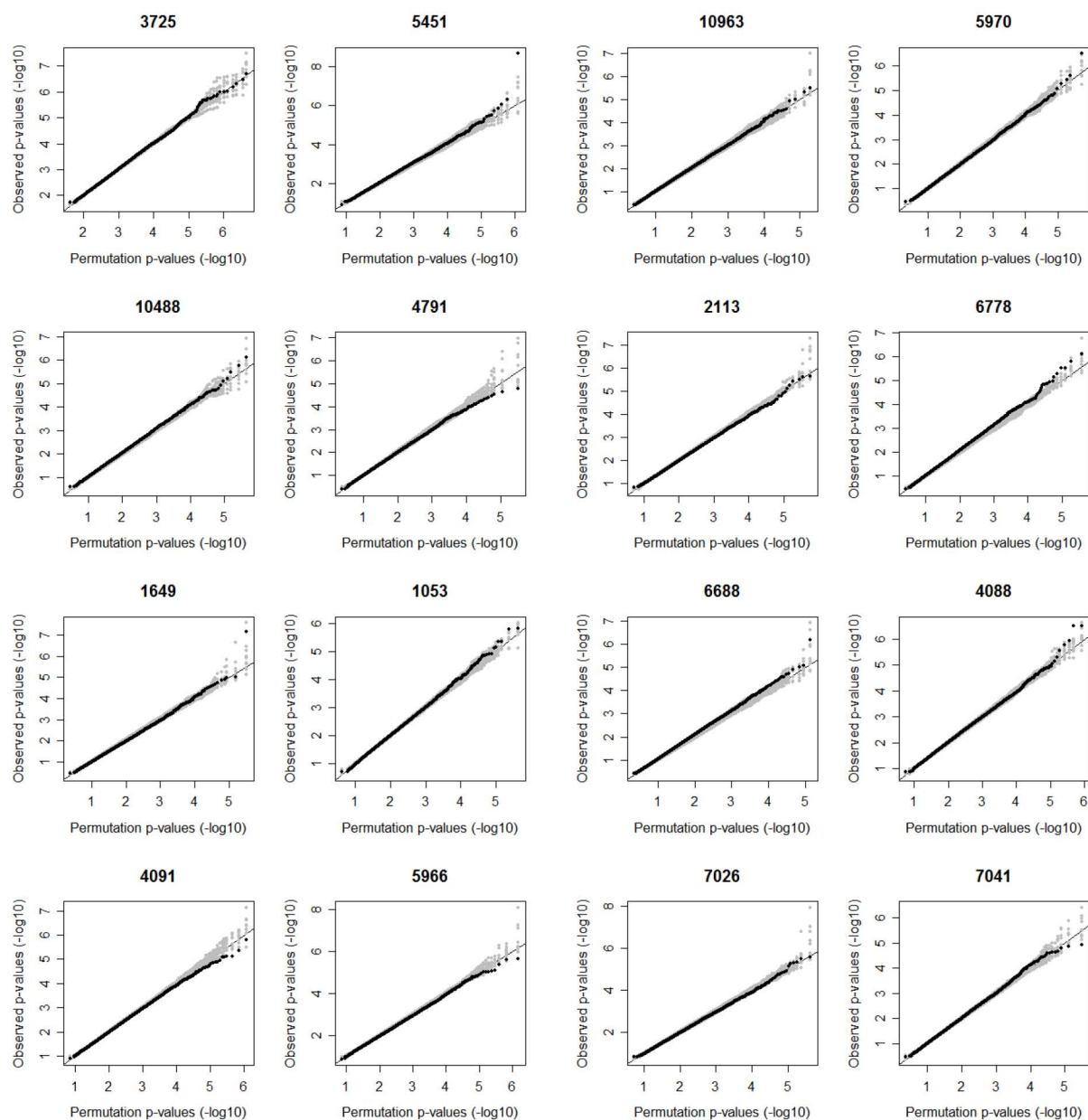

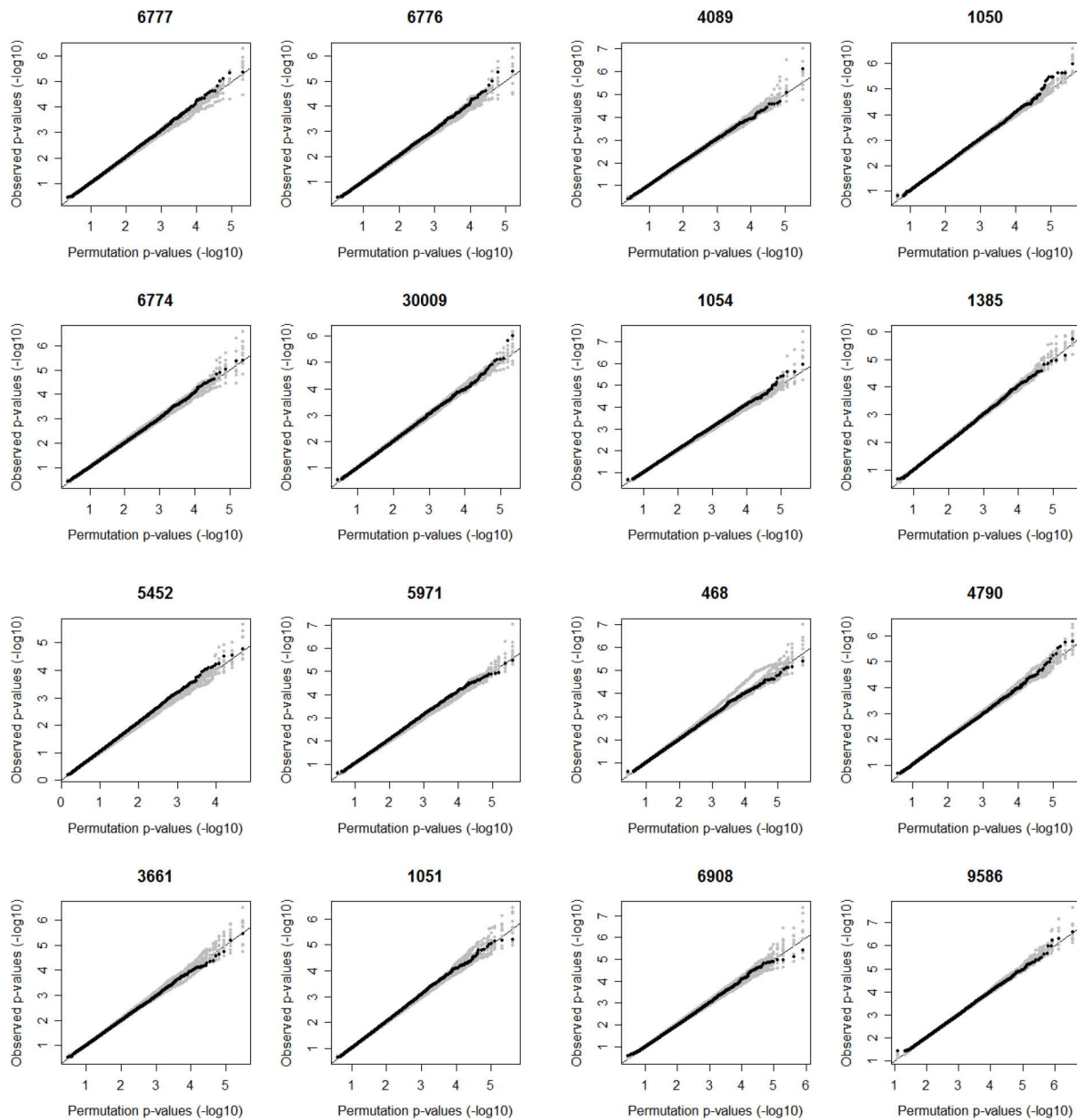

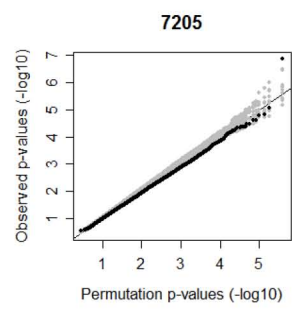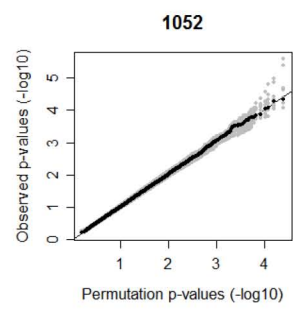

Supplement: Figure S4 — Observed p-values for association between genotype and log-fold change in expression (GC/control) are plotted against p-values from permutations, representing expectations under the null, for a) SNPs within 500 kb (only SNP with minimum p-value is plotted for each gene) of the gene that encodes the GR and b) SNPs within 500 kb of transcription factors known to interact with the GR. c) SNPs within 500 kb of each of the interacting transcription factors and d) within 100 kb of each of the interacting transcription factors. (PDF) [file pgen.1002162.s004.pdf]

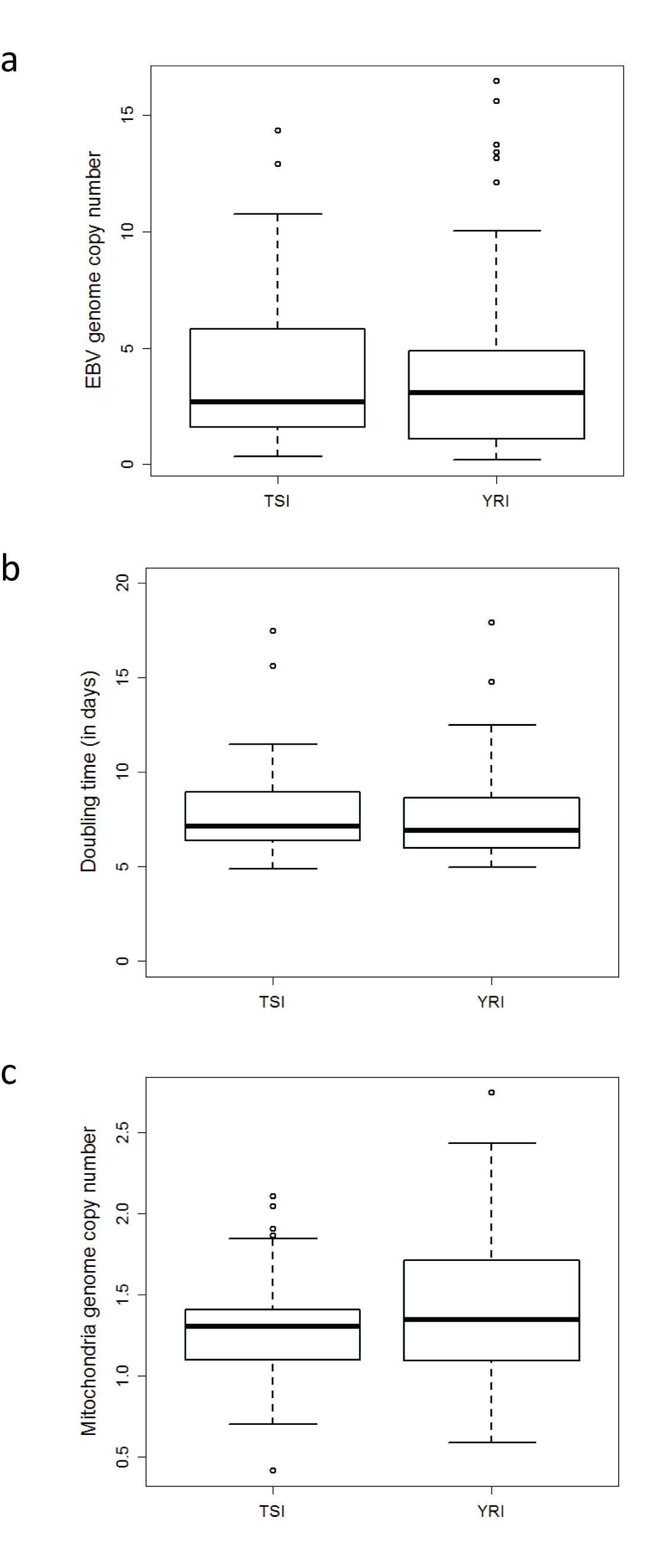

Supplement: Figure S5 — Differences between TSI and YRI LCLs in the distribution of factors known to affect LCL biology. We do not observe significant differences in a) EBV genome copy number (p = 0.824) or b) growth rate (p = 0.477). c) We did observe a significantly higher level of mitochondrial genome copy number among YRI LCLs (p = 0.0463). (TIF) [file pgen.1002162.s005.tif]

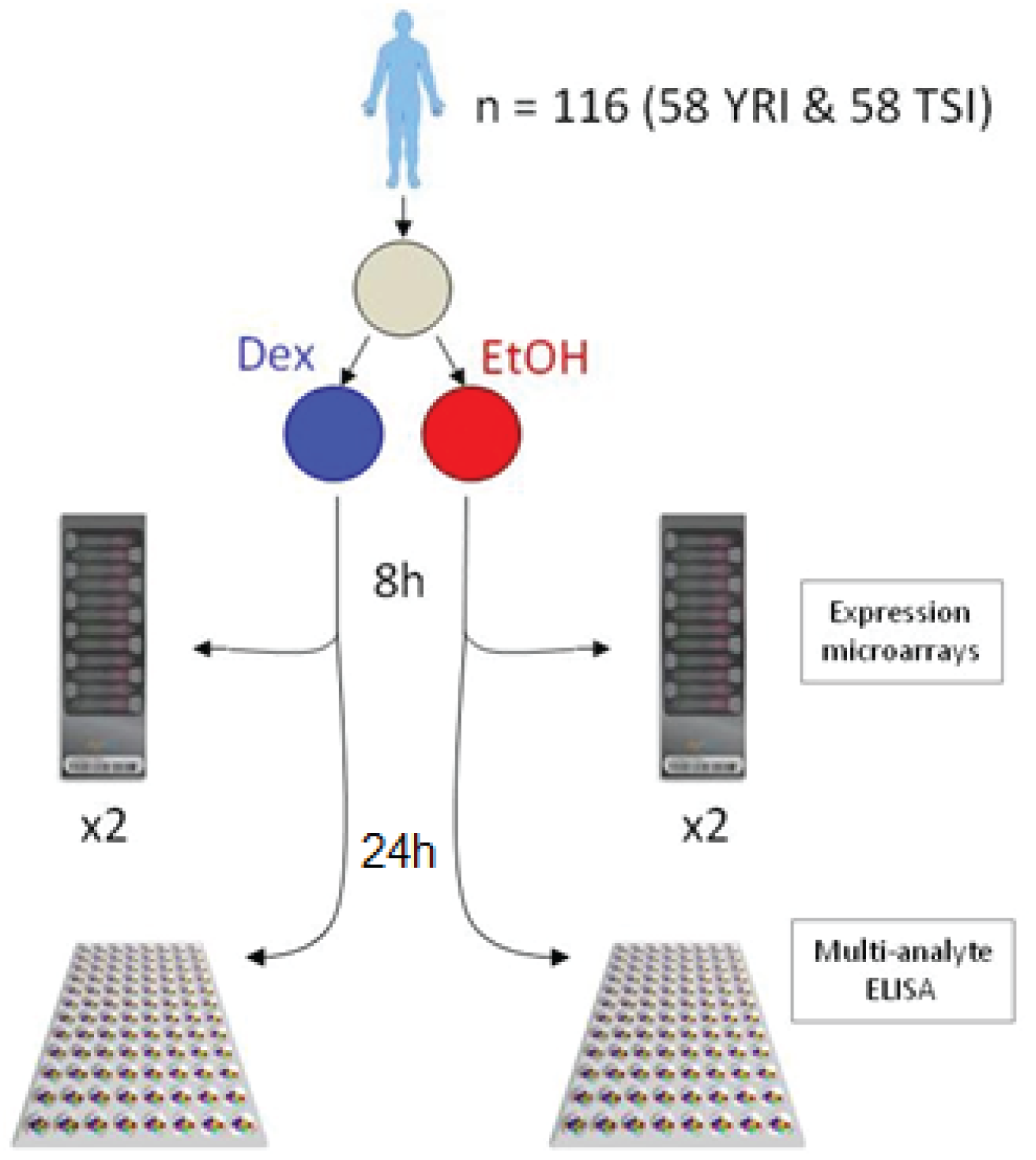

Supplement: Figure S6 — The study design used in this experiment is shown. For each of 116 LCLs, one aliquot was treated with the synthetic GC dexamethasone and another aliquot was treated with the vehicle for dexamethasone (EtOH) as a treatment control. Two sets of paired aliquots were treated for each LCL, one for 8 hours and the other for 24 hours. RNA was extracted from aliquots treated for 8 hours and hybridized to two replicate arrays (for a total of 4 arrays hybridized per LCL). Supernatant from the aliquots treated for 24 hours were used to assay protein secretion. (TIF) [file pgen.1002162.s006.tif]

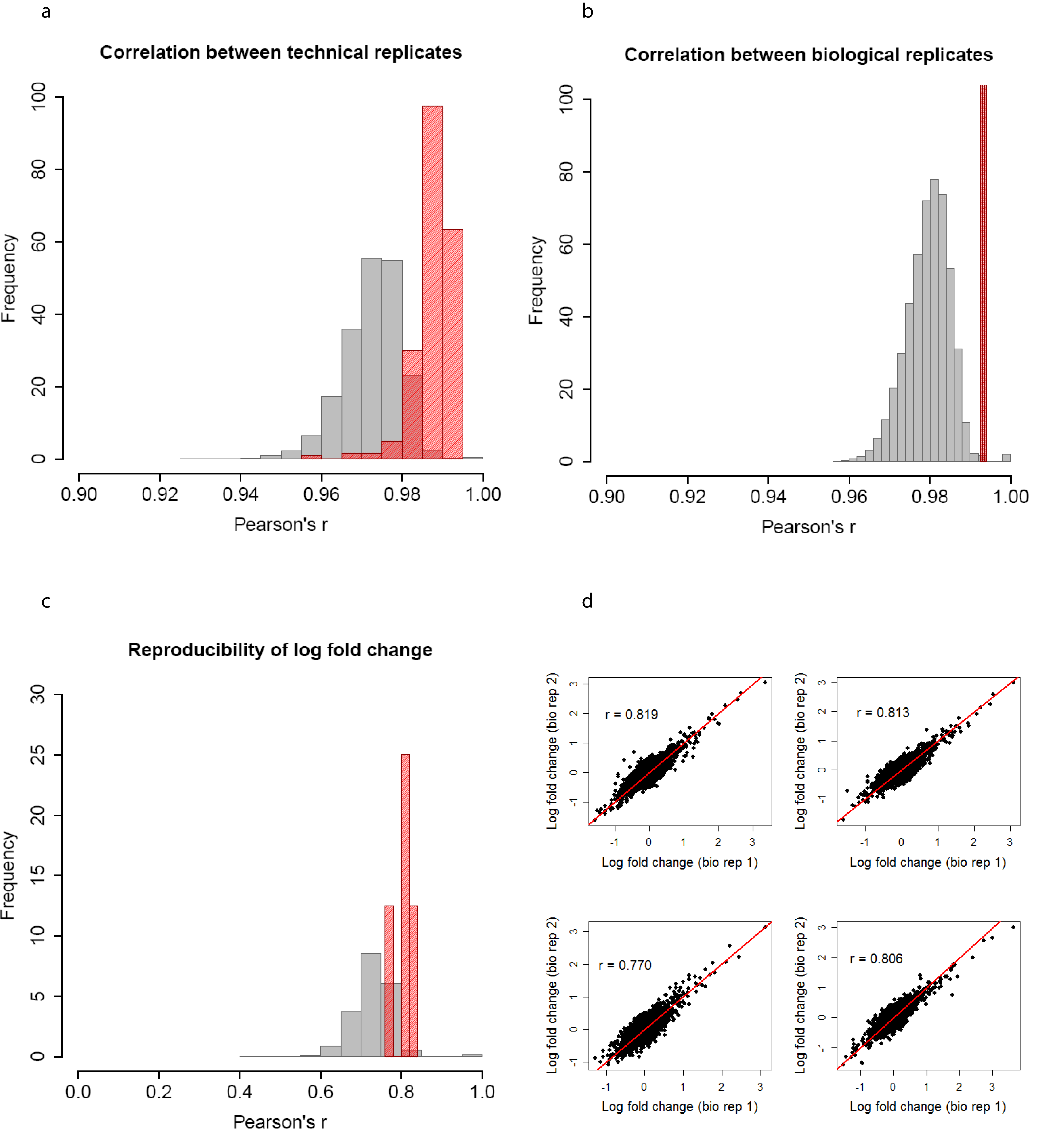

Supplement: Figure S7 — a) Pair-wise correlations between technical replicates (red), representing duplicate RNA hybridizations, tend to be larger than correlations between randomly drawn pairs of arrays (grey), suggesting a limited contribution from RNA hybridization to variation in these measurements. b) Pair-wise correlations of expression levels between biological replicates are always larger (red) than randomly drawn pairs of LCLs (grey), similarly suggesting that variation in cell culturing and treatment protocols described here contribute little to variation in expression measurements. c-d) Log-fold changes (GC/control) across the 4,568 differentially expressed genes compared between biological replicates to assess the reproducibility of response. c) Correlations between biological replicates (red) are higher than expected when comparing randomly drawn pairs of LCLs (grey), suggesting that variation from cell culturing and treatment does not explain the majority of variation in response between LCLs. d) Correlations between replicate pairs are shown for transcriptional response across differentially expressed genes. (TIFF) [file pgen.1002162.s007.tif]

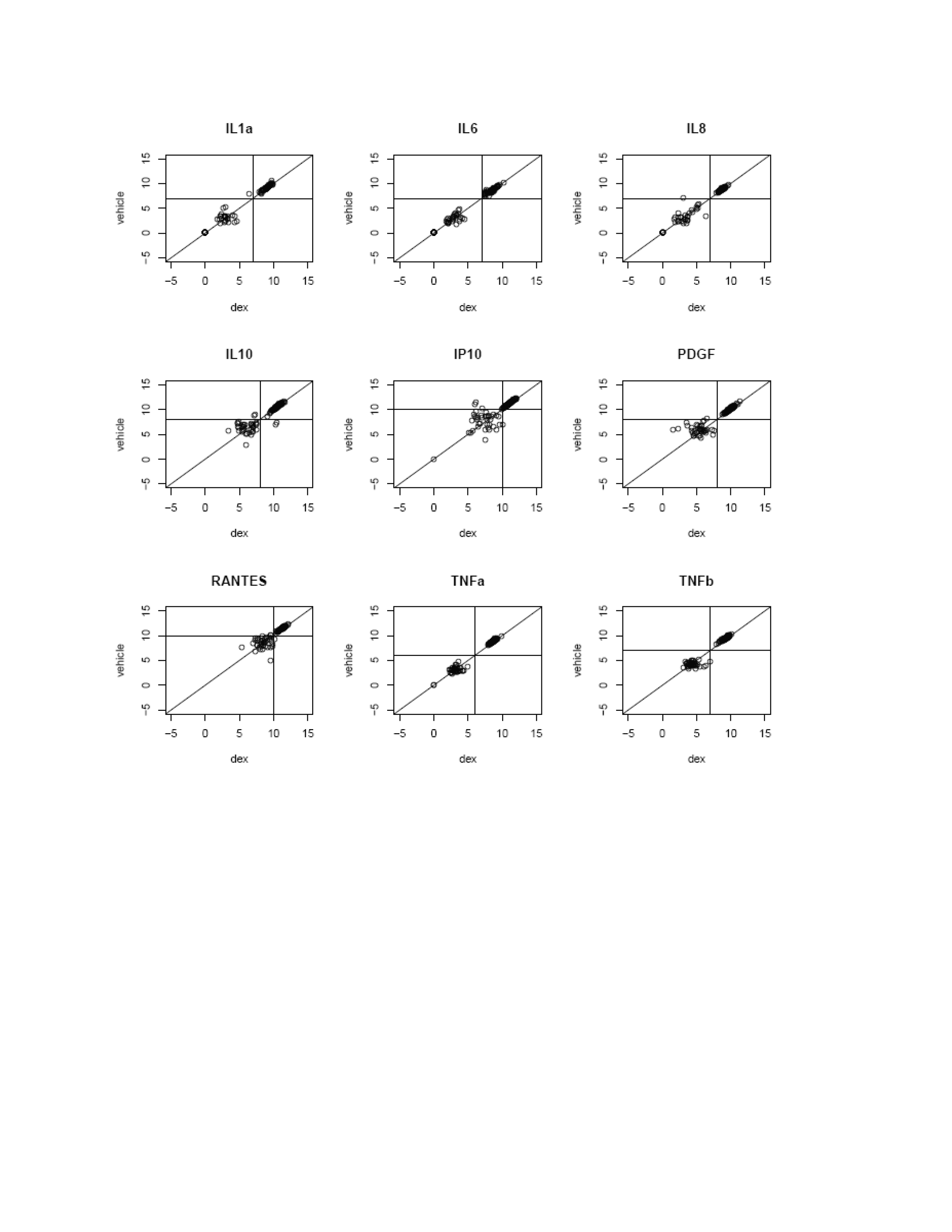

Supplement: Figure S8 — Correlation between secretion levels across individuals for each protein. Secretion levels represent log-transformed ELISA measurements of protein quantity. Horizontal and vertical lines in each plot indicate the threshold used to identify meaningful secretion measurements. (TIF) [file pgen.1002162.s008.tif]

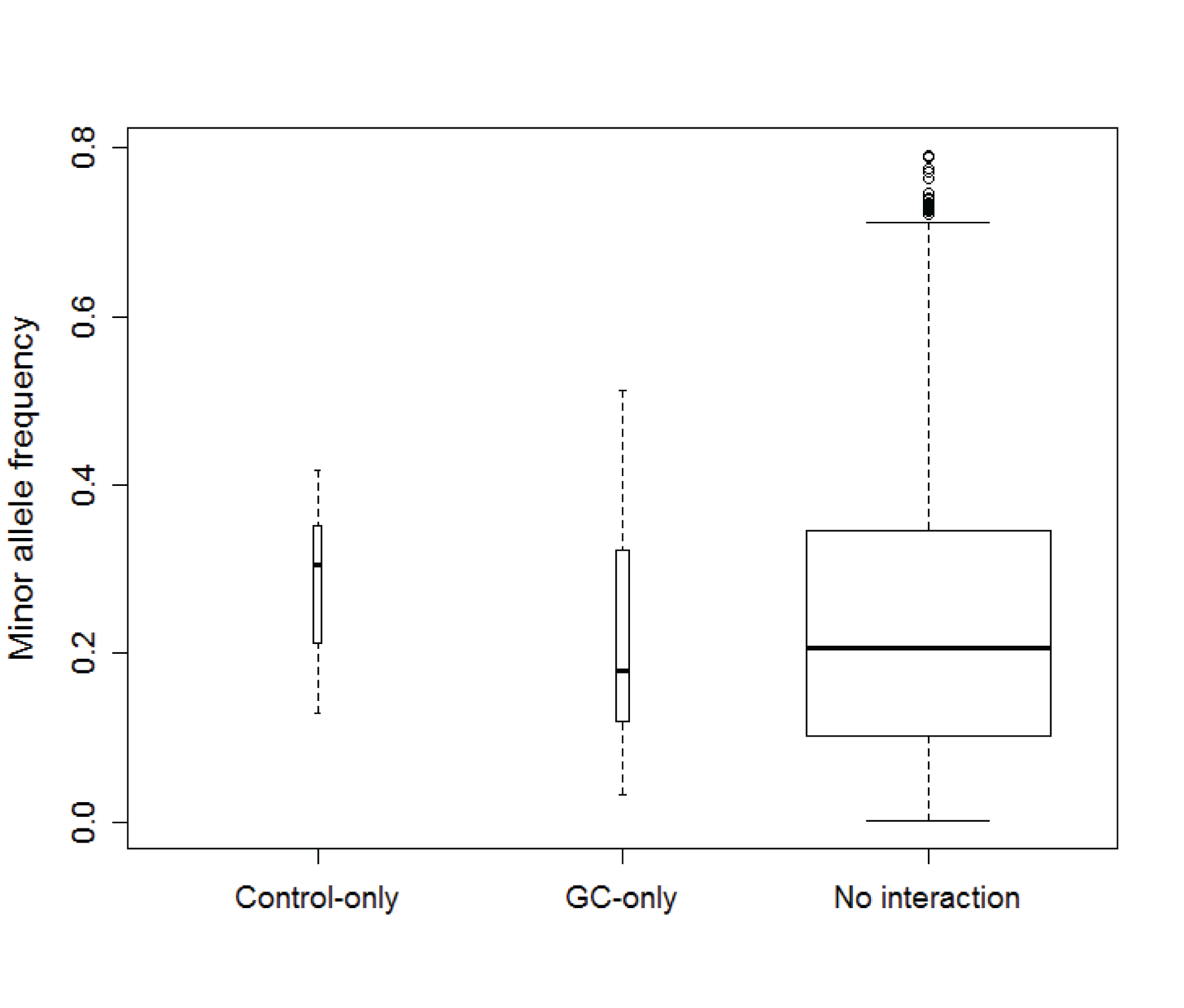

Supplement: Figure S9 — Distribution of minor allele frequency for each candidate eQTN categorized by model. No significant difference is observed, based on Mann-Whitney U test, between control-only and GC-only eQTNs (p = 0.26), GC-only and no-interaction eQTNs (p = 0.94), or control-only and no-interaction eQTNs (p = 0.19). (TIF) [file pgen.1002162.s009.tif]

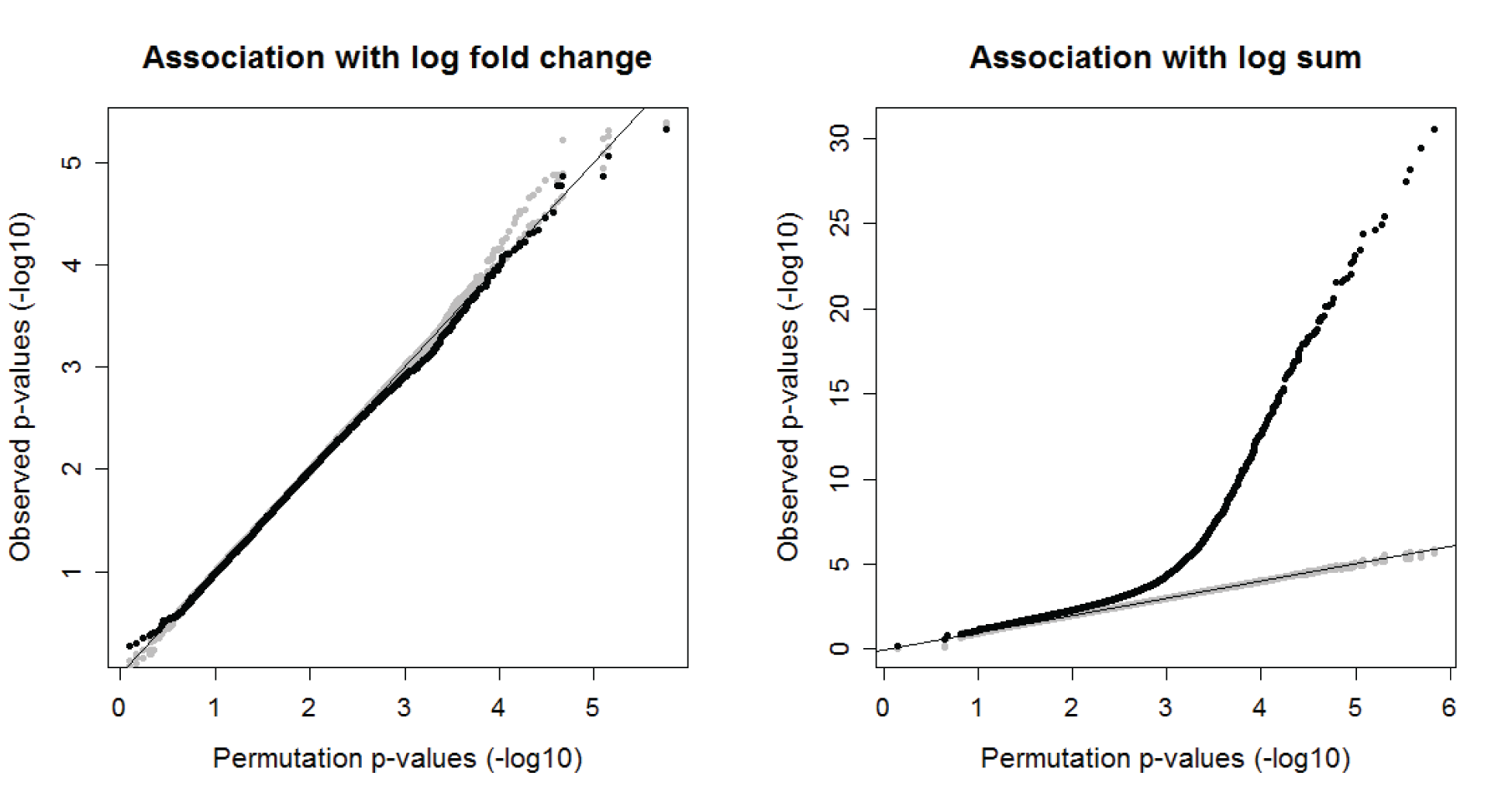

Supplement: Figure S10 — Quantile-quantile plots showing the distribution of p-values from mapping the log fold change and log sum (sum of log expression values in GC-treated and control-treated samples) at genes affected by no-interaction eQTLs. No deviation from null expectations are observed for association with log fold change, while a very strong deviation from expectations under the null is observed for association with the log sum. This is consistent with the stable effect of eQTL genotype on expression at these genes. SNPs within 100 kb were tested against log fold change at each gene. Observed minimum p-values per gene are shown as black dots. Minimum p-values from permutations are shown as grey dots. (TIF) [file pgen.1002162.s010.tif]
