# Supplementary material for: Interactions between Glucocorticoid Treatment and Cis-Regulatory Polymorphisms Contribute to Cellular Response Phenotypes
Source: PLoS Genet. 2011 Jul 7;7(7):e1002162. doi: 10.1371/journal.pgen.1002162 (PMC3131293; doi:10.1371/journal.pgen.1002162)
Supplement: Table S1 — Association between gene expression and EBV copy number, mitochondrial copy number, and growth rate. (PDF) [file pgen.1002162.s011.pdf]

	Number of associated genes (FDR<0.1)	
	Expression in control-treated samples	Log-fold change in expression
Growth rate	3,529	36
Mitochondria genome copy number	2,306	9
EBV genome copy number	578	2
