# Supplementary material for: Interactions between Glucocorticoid Treatment and Cis-Regulatory Polymorphisms Contribute to Cellular Response Phenotypes
Source: PLoS Genet. 2011 Jul 7;7(7):e1002162. doi: 10.1371/journal.pgen.1002162 (PMC3131293; doi:10.1371/journal.pgen.1002162)
Supplement: Table S2 — eQTLs that interact with GC treatment. (PDF) [file pgen.1002162.s012.pdf]

Gene name	Gene ID	Most likely model	Posterior probability of eQTL following most likely model	Most associated SNP	Minor allele frequency of most associated SNP	Marginal evidence of association ($\log_{10}$ Bayes factor)	Genotypic effect on log fold change†	P-value from mapping log fold change
C9orf5	23731	GC-only	1.00	rs10816772	0.31	9.79	0.79	1.23×10^{-9}
MFGE8	4240	GC-only	1.00	rs3784752	0.15	6.18	0.90	2.16×10^{-7}
LSG1	55341	GC-only	1.00	rs898820	0.43	10.02	0.61	5.92×10^{-7}
C1orf106	55765	GC-only	1.00	rs4915463	0.51	9.21	0.63	9.86×10^{-9}
AIRE	326	GC-only	0.99	rs743479	0.19	4.17	-0.44	2.33×10^{-3}
MS4A7	58475	GC-only	0.99	rs2233252	0.40	4.59	0.66	2.44×10^{-7}
BIRC3	330	GC-only	0.98	rs11602147	0.39	4.23	0.70	2.82×10^{-7}
TNIP1	10318	GC-only	0.97	rs6870205	0.12	3.70	0.88	3.08×10^{-6}
NQO1	1728	GC-only	0.92	rs689459	0.10	12.27	1.15	1.34×10^{-8}
LONP1	9361	GC-only	0.92	rs8104044	0.24	3.34	-0.51	4.22×10^{-4}
NLRP2	55655	GC-only	0.90	rs12104320	0.03	3.82	-1.03	2.02×10^{-3}
GSTM3	2947	GC-only	0.87	rs6537874	0.10	4.23	0.75	2.18×10^{-4}
CST7	8530	GC-only	0.85	rs4610125	0.12	3.70	-1.10	3.19×10^{-5}
*HSD11B1L**	374875	GC-only	0.83	rs3745640	0.25	2.88	-0.40	4.95×10^{-3}
FAM134B	54463	GC-only	0.83	rs31287	0.32	5.08	0.52	2.11×10^{-5}
SGK1	6446	GC-only	0.78	rs4429966	0.15	2.52	-0.50	0.012
PDGFRL	5157	GC-only	0.77	rs2517202	0.16	3.42	0.50	1.21×10^{-3}
PANK3	79646	GC-only	0.71	rs7734906	0.08	2.89	-0.73	1.44×10^{-3}
MT1X	4501	control-only	1.00	rs7195394	0.42	6.10	0.71	6.11×10^{-8}
*EDF1**	8721	control-only	0.99	rs4880172	0.29	4.05	0.04	0.77
FBXL6	26233	control-only	0.95	rs10448143	0.14	3.48	0.63	1.26×10^{-4}
SRD5A2	6716	control-only	0.94	rs11690596	0.30	3.40	0.48	4.23×10^{-4}
*CSNK1E**	1454	control-only	0.88	rs5750597	0.31	3.10	0.23	0.093
SYT17	51760	control-only	0.80	rs229020	0.35	3.62	-0.48	8.87×10^{-4}
MCFD2	90411	control-only	0.77	rs4953439	0.13	2.43	0.79	5.21×10^{-5}
C12orf45	121053	control-only	0.72	rs10861313	0.36	5.13	0.39	1.03×10^{-3}

* eQTLs with genotypic effects in opposite directions in the YRI and TSI populations.
† Expressed in standard deviations.
