# Supplementary material for: Interactions between Glucocorticoid Treatment and Cis-Regulatory Polymorphisms Contribute to Cellular Response Phenotypes
Source: PLoS Genet. 2011 Jul 7;7(7):e1002162. doi: 10.1371/journal.pgen.1002162 (PMC3131293; doi:10.1371/journal.pgen.1002162)
Supplement: Table S3 — Effects of GC treatment on cytokine secretion. (PDF) [file pgen.1002162.s013.pdf]

Protein	Log-fold change in secretion	p
IL1a	16.03	4.58
IL6	1.51	2.51
RANTES	1.35	1.76
MDC	1.53	1.04
TNFb	0.78	1.16
IL10	0.87	0.73
IL8	0.70	0.80
TNFa	0.59	0.67
IP10	0.57	0.49
