# Supplementary material for: Interactions between Glucocorticoid Treatment and Cis-Regulatory Polymorphisms Contribute to Cellular Response Phenotypes
Source: PLoS Genet. 2011 Jul 7;7(7):e1002162. doi: 10.1371/journal.pgen.1002162 (PMC3131293; doi:10.1371/journal.pgen.1002162)
Supplement: Table S4 — Maximum likelihood estimates of the proportion of genes with an association following each model for all the predictor variables we tested against gene expression. (PDF) [file pgen.1002162.s014.pdf]

Predictor	π_0	Model probabilities (conditional on effect)			
		GC-only	control-only	unequal	No interaction
Cis SNPs (eQTL mapping)	0.207 (0.181,0.232)	0.031 (0.021,0.042)	0.026 (0.016,0.038)	0.001 (0.000,0.003)	0.942 (0.927,0.955)
Population differences	0.5834 (0.569,0.597)	0.090 (0.076,0.105)	0.089 (0.075,0.104)	0.009 (0.002,0.019)	0.812 (0.793,0.830)
Growth rate	0.6339 (0.623,0.645)	0.001 (0,0.004)	0.022 (0.016,0.029)	0.000 (0.000,0.001)	0.977 (0.970,0.983)
Mitochondrial genome copy number	0.7874 (0.777,0.796)	0.007 (0.003,0.014)	0.01 (0.005,0.017)	0.000 (0.000,0.001)	0.983 (0.974,0.990)
EBV genome copy number	0.8725 (0.865,0.881)	0.004 (0,0.013)	0.006 (0,0.013)	0.000 (0.000,0.003)	0.991 (0.980,0.998)

Confidence intervals for each point estimates are shown in parentheses.
