# Supplementary material for: Interactions between Glucocorticoid Treatment and Cis-Regulatory Polymorphisms Contribute to Cellular Response Phenotypes
Source: PLoS Genet. 2011 Jul 7;7(7):e1002162. doi: 10.1371/journal.pgen.1002162 (PMC3131293; doi:10.1371/journal.pgen.1002162)
Supplement: Table S5 — SNPs showing strongest signal for interaction from the SNP-based Bayesian analysis. (PDF) [file pgen.1002162.s015.pdf]

Supplementary Table 5. SNPs showing strongest signal for interaction from SNP-based Bayesian analysis*

SNP	gene	log10 (BF int)**	log10 (BF model 3)	log10 (BF model 2)	log10 (BF model 4)	log10 (BF model 1)
rs17154274	55973	4.4	4.4	4.3	4.3	-0.5
rs16872355	55973	4.4	4.4	4.3	4.3	-0.5
rs10901769	64077	4.4	2.3	5.5	4.8	1.1
rs1217973	3660	4.3	4.3	2.4	3.6	-0.2
rs9816963	8971	4.3	4.3	4.2	3.9	-0.8
rs3806489	57590	4.2	2.6	4.3	3.5	0.1
rs959987	121260	4.1	2.8	4.1	3.3	-0.3
rs10734965	121260	4.1	2.9	4.1	3.3	-0.4
rs959988	121260	4.1	2.9	4.1	3.3	-0.4
rs13261781	57210	4.0	4.0	0.9	3.4	0.0
rs3736430	4214	4.0	1.9	4.7	3.9	0.7
rs10773580	121260	3.9	2.8	3.9	3.2	-0.4
rs2115794	84068	3.9	3.9	1.3	2.9	0.0
rs296827	50808	3.9	3.9	3.1	3.2	-0.5
rs13016817	150468	3.9	3.9	2.6	3.2	0.0
rs13015273	150468	3.9	3.9	2.6	3.2	0.0
rs3862663	27339	3.9	3.0	3.9	3.4	-0.4
rs10773579	121260	3.9	2.6	3.9	3.0	-0.4
rs542380	23649	3.9	3.1	3.9	3.1	-0.6
rs658181	28970	3.9	9.3	1.3	9.3	5.4
rs586293	28970	3.9	9.3	1.3	9.3	5.4
rs625249	28970	3.9	9.3	1.3	9.3	5.4
rs652621	28970	3.9	9.3	1.3	9.3	5.4
rs655158	28970	3.9	9.3	1.3	9.3	5.4
rs3741613	121260	3.9	2.7	3.9	3.0	-0.4
rs6884959	54974	3.8	4.5	2.4	3.8	0.7
rs17585837	3660	3.8	3.8	2.8	3.3	-0.6
rs1904274	57733	3.8	0.4	3.9	2.9	0.1

rs2618954	57733	3.8	0.4	3.9	2.9	0.1
rs585713	28970	3.8	9.3	1.3	9.4	5.5
rs1428919	28970	3.8	9.1	1.2	9.2	5.3
rs2170989	121260	3.8	2.5	3.8	2.7	-0.5
rs16988011	55954	3.7	2.0	3.7	3.3	-0.1
rs7839	50808	3.7	3.7	2.8	3.1	-0.3
rs10168212	8291	3.7	0.2	4.1	3.7	0.3
rs13185706	309	3.7	2.1	3.7	3.0	-0.1
rs2399797	8872	3.7	4.6	0.9	3.6	0.9
rs4783009	23406	3.7	3.7	1.8	3.2	0.0
rs611315	50808	3.7	3.7	2.8	2.9	-0.5
rs1627672	131583	3.7	1.9	3.7	2.5	-0.6
rs1675951	131583	3.7	1.9	3.7	2.5	-0.6
rs1773194	131583	3.7	1.9	3.7	2.5	-0.6
rs1320622	131583	3.7	1.9	3.7	2.4	-0.6
rs1705971	131583	3.7	1.9	3.7	2.4	-0.6
rs1705967	131583	3.7	1.9	3.7	2.4	-0.6
rs6923993	11329	3.7	-0.1	8.3	7.9	4.7
rs247826	23406	3.7	3.7	2.7	3.7	0.0
rs7118900	54970	3.7	4.5	1.4	3.7	0.8
rs1025734	57590	3.6	2.0	3.8	3.0	0.2
rs10071628	54974	3.6	4.1	2.2	3.3	0.4
rs10849829	64897	3.6	3.6	2.4	2.5	-0.7
rs9930179	10200	3.6	1.6	3.8	2.8	0.2
rs1182933	64897	3.6	4.6	2.0	4.2	1.0
rs529251	54956	3.6	3.6	1.1	3.2	-0.5
rs2077557	84520	3.6	3.8	1.8	2.9	0.2
rs7744363	11329	3.6	-0.1	8.1	7.7	4.5
rs2605328	552889	3.6	1.8	3.6	2.8	-0.2
rs16988049	55954	3.6	2.4	3.6	3.0	-0.6
rs1037928	131583	3.6	1.8	3.6	2.4	-0.6

rs1627849	131583	3.6	1.8	3.6	2.4	-0.6
rs12051269	23406	3.6	3.6	2.5	3.2	-0.4
rs7513645	64710	3.6	1.2	3.6	2.7	-0.1
rs6927461	6941	3.5	0.3	3.9	3.1	0.4
rs7120629	80071	3.5	1.3	3.7	3.0	0.2
rs12622285	150962	3.5	1.4	3.6	3.3	0.0
rs4760533	121260	3.5	2.1	3.5	2.5	-0.4
rs1543852	760	3.5	3.5	2.3	2.7	-0.2
rs3739558	29988	3.5	2.5	3.5	2.6	-0.3
rs703971	57178	3.5	3.4	3.5	2.9	-0.8
rs10847694	121260	3.5	2.3	3.5	2.5	-0.5
rs11752262	7919	3.5	3.5	0.9	2.6	-0.1
rs8001869	122258	3.5	0.4	3.7	3.6	0.2
rs10847693	121260	3.5	2.2	3.5	2.5	-0.5
rs7112607	80071	3.5	1.3	3.7	3.0	0.2
rs10498962	8732	3.5	3.5	2.8	2.9	-0.4
rs11827270	80071	3.5	1.4	3.7	3.0	0.2
rs2113469	2960	3.5	2.0	3.5	3.3	-0.7
rs1432298	150962	3.5	1.4	3.6	3.3	0.1
rs17054946	5550	3.5	1.6	3.7	3.2	0.2
rs12816551	121260	3.5	2.2	3.5	2.4	-0.5
rs1675953	131583	3.5	1.6	3.5	2.3	-0.5
rs7974764	121260	3.5	2.2	3.5	2.4	-0.5
rs12170399	266747	3.5	2.3	3.5	3.2	-0.9
rs12167140	266747	3.5	2.3	3.5	3.2	-0.9
rs10848775	7004	3.5	3.0	3.5	2.8	-0.5
rs9680742	266747	3.5	2.3	3.5	3.2	-0.9
rs2260399	64897	3.5	3.5	2.1	2.3	-0.6
rs11059922	121260	3.5	2.2	3.5	2.4	-0.5
rs8024741	54956	3.5	3.5	0.6	2.8	-0.4
rs11823396	80071	3.5	1.7	3.5	2.9	-0.1

rs3739557	29988	3.4	2.4	3.4	2.5	-0.3
rs531647	57590	3.4	1.9	3.8	3.2	0.4
rs7211959	11056	3.4	2.4	3.4	3.1	-0.2
rs1675947	131583	3.4	1.5	3.4	2.2	-0.4
rs644009	28970	3.4	7.5	1.4	7.6	4.1
rs12040674	6913	3.4	2.9	3.4	3.1	0.0
rs4835264	152485	3.4	2.5	3.4	2.9	-0.3
rs13143990	201895	3.4	2.8	3.4	2.5	-0.6
rs655699	28970	3.4	7.5	1.4	7.5	4.1
rs11020522	28970	3.4	7.5	1.4	7.5	4.1
rs673869	28970	3.4	7.5	1.4	7.5	4.1
rs4711453	11329	3.4	-0.1	9.1	9.0	5.7
rs7089060	80019	3.4	-0.3	3.9	2.6	0.5
rs1570368	11329	3.4	-0.2	9.1	9.1	5.8
rs7075656	80019	3.4	0.0	3.9	2.9	0.5
rs2515938	8602	3.4	3.4	1.3	2.4	-0.3
rs11065385	64897	3.4	5.0	1.7	4.7	1.6
rs4963166	51286	3.3	3.7	2.1	3.6	0.3
rs12159277	55954	3.3	2.3	3.3	2.8	-0.6
rs17408310	952	3.3	3.3	2.9	2.5	-0.8
rs12035633	7159	3.3	1.4	3.4	2.3	0.1
rs4670676	10153	3.3	1.7	3.5	2.8	0.2
rs8179426	6913	3.3	2.8	3.3	3.0	-0.1
rs17107517	54708	3.3	1.0	3.3	2.2	-0.6
rs480829	57590	3.3	1.8	3.6	3.0	0.3
rs12569041	6913	3.3	2.8	3.3	3.0	-0.1
rs1183910	64897	3.3	4.8	1.6	4.3	1.5
rs4858802	6599	3.3	3.4	2.4	3.2	0.1
rs4766480	326625	3.3	3.3	1.7	3.1	-0.7
rs10187805	1286	3.3	3.5	-0.1	2.3	0.2
rs801089	673	3.3	3.5	0.5	2.8	0.2

rs10817453	1318	3.3	0.9	3.8	2.9	0.5
rs1615164	23247	3.3	3.6	0.0	2.6	0.3
rs10055619	23107	3.3	2.6	3.3	2.2	-0.9
rs6982826	56943	3.3	3.3	0.7	2.2	-0.4
rs7008687	56943	3.3	3.3	0.7	2.2	-0.4
rs11076172	9709	3.3	1.9	3.3	2.4	-0.2
rs10402974	126003	3.3	3.3	2.3	2.7	-0.1
rs7954766	326625	3.2	3.2	1.7	3.1	-0.6
rs6659097	148641	3.2	1.7	3.2	3.1	0.0
rs427982	126205	3.2	2.9	3.2	2.9	-0.8
rs2920351	6397	3.2	3.2	2.6	2.4	-0.7
rs17104512	10490	3.2	3.2	1.8	2.9	-0.1
rs10144907	10490	3.2	3.2	1.8	2.9	-0.1
rs983492	121260	3.2	2.1	3.2	2.2	-0.6
rs12516527	6940	3.2	0.6	3.2	3.2	-0.1
rs10134090	10490	3.2	3.2	1.8	2.9	-0.1
rs10131884	10490	3.2	3.2	1.8	2.9	-0.1
rs10140733	10490	3.2	3.2	1.8	2.9	-0.1
rs7145931	2342	3.2	2.2	3.2	2.7	-0.5
rs4965778	79705	3.2	3.2	1.3	2.2	-0.3
rs1551480	23303	3.2	3.2	1.4	2.7	0.0
rs10494111	2949	3.2	0.5	3.2	3.8	-0.1
rs10882037	54708	3.2	1.0	3.2	2.2	-0.6
rs10883465	55280	3.2	3.2	1.4	2.5	-0.4
rs13269737	57210	3.2	3.2	0.6	2.5	-0.3
rs13270024	57210	3.2	3.2	0.6	2.5	-0.3
rs9787608	54708	3.2	1.0	3.2	2.2	-0.6
rs10040353	54974	3.2	4.1	1.9	3.6	0.9
rs6880381	54974	3.2	4.1	1.9	3.6	0.9
rs9926856	79447	3.2	2.3	3.2	2.7	-0.4
rs3765701	49856	3.2	3.2	1.7	2.1	-0.6

rs194936	55603	3.2	3.2	3.2	2.5	-0.6
rs7849	55280	3.2	3.2	1.6	2.5	-0.5
rs16924480	966	3.2	1.6	3.5	3.0	0.3
rs12442723	4643	3.2	3.3	0.7	2.4	0.1
rs4961155	8767	3.2	1.5	3.5	2.8	0.3
rs1609570	654	3.2	2.3	3.2	2.2	-0.7
rs5997520	55954	3.2	2.6	3.2	3.0	-0.4
rs2708104	64897	3.2	3.2	1.4	2.1	-0.2
rs1951565	9056	3.2	3.2	3.2	2.7	-0.4
rs12158466	55954	3.2	2.6	3.2	3.1	-0.3
rs2599704	137682	3.2	1.0	3.2	2.1	0.0
rs1214589	57559	3.2	3.2	1.9	2.2	-0.6
rs1561103	10801	3.2	1.1	3.7	2.9	0.5
rs11220305	219844	3.2	1.8	3.2	2.5	-0.1
rs1147603	57559	3.2	3.2	1.5	2.1	-0.5
rs7992158	122258	3.2	-0.8	3.5	2.5	0.3
rs12576535	219844	3.2	1.7	3.2	2.3	-0.2
rs11818932	8872	3.2	3.4	1.4	2.8	0.3
rs6537501	170371	3.2	3.2	3.1	2.1	-1.1
rs4390574	1535	3.2	2.1	3.2	2.4	-0.5
rs7841178	760	3.2	3.2	1.9	2.3	-0.1
rs11071835	54956	3.2	3.2	0.5	2.5	-0.5
rs11985733	760	3.2	3.2	1.9	2.3	-0.1
rs2285604	147694	3.1	0.4	3.1	2.1	-0.1
rs4083591	9213	3.1	3.1	0.9	2.2	-0.2
rs4917774	80019	3.1	-0.2	3.4	2.3	0.3
rs3845406	9213	3.1	3.1	1.7	2.5	-0.6
rs4919105	80019	3.1	-0.1	3.4	2.3	0.3
rs17277872	9056	3.1	3.1	2.8	2.5	-0.4
rs4899215	10490	3.1	3.1	1.6	2.7	0.0
rs602060	9328	3.1	1.8	3.1	2.0	-0.9

rs17277879	9056	3.1	3.1	2.8	2.5	-0.4
rs17029376	54626	3.1	3.1	1.7	2.9	0.0
rs740116	55954	3.1	2.2	3.1	2.4	-0.9
rs6556177	54974	3.1	3.6	1.7	2.9	0.5
rs954439	3437	3.1	2.3	3.1	2.2	-0.6
rs11869519	91607	3.1	2.0	3.1	2.3	-0.2
rs2022449	7292	3.1	3.0	3.1	2.2	-0.9
rs14490	56267	3.1	2.2	3.1	2.4	-0.4
rs4281692	64131	3.1	3.1	1.5	2.1	-0.2
rs10981670	1318	3.1	0.8	3.5	2.6	0.4
rs17183465	326625	3.1	3.1	1.4	2.7	-0.7
rs3742014	326625	3.1	3.1	1.4	2.8	-0.7
rs6999170	56943	3.1	3.1	0.8	2.1	-0.4
rs2244608	64897	3.1	3.7	1.4	3.1	0.6
rs11908683	55741	3.1	3.1	2.3	2.6	-0.7
rs1390247	7326	3.1	3.1	1.5	2.3	0.0
rs331677	7409	3.1	1.2	3.1	2.7	-0.4
rs12627866	55954	3.1	1.8	3.1	2.3	-0.7
rs9955228	26256	3.1	4.4	0.7	3.7	1.3
rs6942989	84310	3.1	1.4	3.1	2.2	-0.2
rs4290258	85363	3.1	1.4	3.1	2.2	-0.1
rs12001239	9933	3.1	2.4	3.1	2.5	-0.6
rs11259158	83641	3.1	1.5	3.1	2.2	-0.2
rs2259883	64897	3.1	3.1	1.3	2.2	0.1
rs6759981	60526	3.1	3.1	1.2	2.7	0.0
rs16988062	55954	3.1	2.0	3.1	2.6	-0.4
rs1169300	64897	3.1	3.8	1.2	3.1	0.7
rs1832775	64710	3.1	0.8	3.2	2.3	0.1
rs8137495	266747	3.1	1.5	3.1	2.5	-0.7
rs11214601	54970	3.1	3.1	2.0	2.5	-0.2
rs9324301	122258	3.1	-0.8	3.4	2.4	0.4

rs10981669	1318	3.1	0.8	3.5	2.6	0.4
rs4452723	373156	3.1	3.1	0.6	1.9	0.0
rs7211475	10241	3.0	2.5	3.0	3.5	-0.3
rs10091566	760	3.0	3.0	1.8	2.3	-0.1
rs13171398	309	3.0	1.6	3.0	2.4	-0.1
rs394436	7409	3.0	1.0	3.0	2.5	-0.4
rs16837370	128229	3.0	0.8	3.0	2.7	-0.6
rs4683182	1230	3.0	2.8	3.0	2.8	-0.4
rs17135316	25823	3.0	-0.4	3.0	2.2	0.0
rs16988048	60526	3.0	3.1	1.2	2.6	0.1
rs1996500	137682	3.0	1.0	3.0	1.9	-0.1
rs160665	56897	3.0	3.0	0.9	2.0	-0.4
rs7181866	2553	3.0	2.8	3.0	3.2	-0.3
rs8108375	22933	3.0	0.6	3.0	2.6	-0.3
rs160668	56897	3.0	3.0	0.9	2.0	-0.4
rs1989455	147694	3.0	0.3	3.0	1.9	0.0
rs17154282	55973	3.0	2.2	3.0	2.5	-0.4
rs10164812	1286	3.0	3.0	0.5	2.3	0.0
rs17104486	10490	3.0	3.0	1.7	2.7	-0.1
rs160664	56897	3.0	3.0	0.7	1.9	-0.2
rs1214550	57559	3.0	3.0	1.7	1.8	-0.8
rs12102635	146330	3.0	3.0	0.6	1.9	-0.2
rs1189402	256764	3.0	3.7	-0.9	3.1	0.7
rs1022549	2762	3.0	3.0	2.0	2.5	-0.1
rs3810911	1318	3.0	0.8	3.5	2.6	0.4
rs703973	57178	3.0	2.7	3.0	2.3	-0.7
rs13018551	57590	3.0	1.6	3.2	2.6	0.2
rs7164113	374618	3.0	2.0	3.0	2.4	-0.1
rs7258292	388524	3.0	0.7	3.0	2.0	-0.2
rs4766613	326625	3.0	3.0	1.5	2.8	-0.7
rs11186860	54708	3.0	0.8	3.0	2.0	-0.6

rs12919746	23406	3.0	3.0	2.2	2.8	-0.4
rs10798331	63931	3.0	3.1	0.4	2.3	0.1

*SNPs in the 26 genes identified with high confidence interactions by the hierarchical model are not included in this table

**BF int = max(BF_2, BF_3, BF_4)/max(BF_1, 1)
