# Supplementary material for: Interactions between Glucocorticoid Treatment and Cis-Regulatory Polymorphisms Contribute to Cellular Response Phenotypes
Source: PLoS Genet. 2011 Jul 7;7(7):e1002162. doi: 10.1371/journal.pgen.1002162 (PMC3131293; doi:10.1371/journal.pgen.1002162)
Supplement: Text S1 — Supplementary Materials and Methods. (PDF) [file pgen.1002162.s016.pdf]

Text S1

Cell culture and dexamethasone treatment

All cellular experiments described were conducted in lymphoblastoid cell lines (LCLs), B lymphocytes immortalized with Epstein-Barr virus, that were collected as a part of the International HapMap project. LCLs from 58 YRI and 58 TSI individuals were obtained from Coriell. Two individuals (one YRI and one TSI) were not included in mapping experiments as genotype data was not available from HapMap. LCLs were thawed and passed once in RPMI media supplemented with 15% fetal bovine serum. LCLs were then washed twice with phosphate-buffered saline and moved to RPMI media supplemented with 15% charcoal-stripped fetal bovine serum. Charcoal stripping dramatically reduces the concentration of hormones, including glucocorticoids. All LCLs were processed with the same lot of charcoal-stripped fetal bovine serum to avoid variation between samples in residual glucocorticoid levels. After one passage in media with charcoal-stripped fetal bovine serum (corresponding to a minimum culturing time of 5 days), four separate aliquots of each LCL were seeded in the evening at a density of $5x10^5$ cells/ml in 10ml of media with 15% charcoal-stripped fetal bovine serum. The following morning, 10^{-4}M dexamethasone (dissolved in 1% ethanol and 99% cell culture media) was added to two aliquots to a final concentration of 10^{-6}M, and an equal amount of vehicle solution (solution composed of 1% ethanol and 99% cell culture media) was added to the other samples as a negative control for treatment. For each LCL, one set of dex and control aliquots was treated for 8 hours (to quantify mRNA abundance) and the other for 24 hours (to assay inflammatory protein secretion). The study design is depicted in Supplementary Fig. 6.

Practical considerations made it infeasible to process large numbers of samples (all time points and treatments) in parallel for each experiment. Therefore, we processed the samples in multiple successive batches. Batch effects (i.e. technical effects that differ between batches) that are confounded with covariates of interest can lead to spurious results [1]. Consequently, LCLs were thawed, cultured and treated in batches completely balanced by treatment, population, technician and time of day. Batch number was recorded for use as a covariate in downstream analyses. Batches were performed in continuous succession over a total time span of no more than six months. If any cell line in a batch failed to grow, collection of the entire batch was repeated. Time of day for RNA extraction, lot of culture medium, and cell culturing protocols were kept constant between samples to minimize technical effects. RNA extraction, processing, and microarray hybridization were also performed in batches completely balanced by treatment, population, technician and time of day.

For quality control purposes, biological replicates were performed for one batch of four cell lines. Specifically, for each of the four LCLs (2 YRI and 2 TSI, 2 males and 2 females), two separate aliquots of frozen cells were thawed, cultured, and treated. In an effort to capture any effects of time on the response, one replicate was performed early in the collection process (1 month after collection began) and the other was collected later (3 months after collection began). Collection of all samples took 4 months.

Measurement of RNA and protein secretion levels

Expression microarrays. For each expression study described in the preliminary data, total RNA was extracted from each cell culture sample using the QIAgen RNeasy Plus mini kit, and was found to be of high quality. RNA was extracted from all 240 samples over the course of 5 days. Total RNA was then reverse transcribed into cDNA, labeled, hybridized to Illumina HumanHT-12 v3 Expression BeadChips and scanned at the Southern California Genotyping Consortium (SCGC: http://scgc.genetics.ucla.edu/) at the University of California at Los Angeles. Each RNA sample was hybridized to two separate arrays (i.e. in two technical replicates). To avoid batch effects on RNA measurements, all 480 microarrays were hybridized within 7 days. Summary data (e.g. mean intensity of each probe across within-array replicates) were obtained using the BeadStudio software (Illumina) at the SCGC.

Quantification of inflammatory markers in the cell culture medium and identification of secretion QTLs that interact with GC treatment. A multianalyte ELISA assay (Millipore) was performed on the culture medium of the cell aliquots treated for 24 hours. The assay was performed at the Flow Cytometry Facility at the University of Chicago, according to the manufacturer instructions. Two technical replicates were run for each sample. Samples were assayed in batches balanced by treatment and population. For each analyte, the average quantity across technical replicates was calculated and used for all subsequent analyses. The correlation structure between paired aliquots for each sample (GC and control) was visually inspected (Supplementary Fig. 8). A small subset of samples with low quantity detected showed no correlation between GC and control aliquots because of noise in the measurement at low concentrations. Consequently, these samples were excluded from downstream analyses. Secretion levels were highly correlated across proteins, likely representing a latent factor that generally affects secretion levels. We sought to map cis-regulatory polymorphisms, which are not expected to affect multiple secreted proteins. To remove the effect of this latent factor, we used linear regression to correct secretion levels at each protein by secretion levels at all other measured proteins. To identify interactions between secretion QTLs and GC treatment, we used the same statistical framework described for mapping eQTL interactions with GC treatment.

Low-level analysis and quality control for microarray data

Low-level microarray analysis was performed using the Bioconductor software package LUMI [2] in R (http://www.r-project.org). Microarray intensity data are often mathematically transformed for the sake of downstream statistical analysis. For example, many statistical tests require that the mean intensity of genes be independent of the variance. It is often the case, however, that the variance increases with the mean in microarray experiments. Log transformation of intensities has traditionally been applied to eliminate this undesirable relationship. We used a similar transformation method, variance stabilizing transformation, which was developed specifically for Illumina BeadChips[3] and has been suggested as the optimal transformation approach for this platform through spike-in experiments conducted by an independent group [4].

Even if a gene is not expressed in a sample, some level of fluorescence will be visible on the array due to non-specific binding. We removed probes with intensities indistinguishable, in all samples, from background fluorescence levels based on control probes that do not map to any known portion of the human genome (at a p-value threshold of 0.01), leaving 23,700 expressed probes for further analyses.

To remove large-scale differences in intensity distributions between arrays, which likely reflect technical effects, we performed quantile normalization across all arrays. This gave all arrays, including arrays from both GC-treated and control-treated samples, the same distribution of intensities while maintaining probe ranks within each array. Array-wise quantile normalization, by definition, removes gross effects of confounders on the mean or variance of arrays' intensity distributions. Additionally, intensities across samples for each probe were quantile normalized to a have a *N(0,1)* distribution to remove the impact of outliers.

Probes were annotated by mapping to the RNA sequences from RefSeq using BLAT. To avoid ambiguity in the source of a signal due to cross-hybridization of similar RNA species, probes that map to multiple Ensembl genes were excluded from further analyses. Probes that contain one or more HapMap SNPs were also removed from further analyses to avoid spurious associations between expression measurements and SNPs in linkage disequilibrium.

Measurement and correction for covariates. Although arrays were quantile normalized to remove the effects of technical factors on the distribution of expression measurements, this approach may fail to remove the effects of confounders if they do not act in a systematic manner across all of the probes on the array. It is, therefore, essential to identify potentially relevant covariates and correct for their effects. Factors such as copies of Epstein Barr virus genome, cellular growth rate and number of mitochondria per cell can affect cellular response to some treatment conditions [5,6]. To avoid spurious results and reduce noise due to these potential confounders, we measured several covariates relevant to LCL biology including: EBV genome copy number, growth rate and mitochondrial genome copy number. Unlike other studies that compared YRI and CEU LCLs [6], we did not observe a significant difference in EBV copy number (p = 0.824 - Supplementary Fig. 5a) or growth rate (p = 0.477 - Supplementary Fig. 5b) between our YRI and TSI LCLs. This validates our choice of the more recently established TSI LCLs as a sample of European ancestry compared to the CEU LCLs. We did, however, find that

mitochondrial genome copy number is significantly higher among YRI LCLs ($p = 0.0463$ – Supplementary Fig. 5c).

We used Bayesian linear regression to identify genes where these factors were associated with the transcriptional response (as described for mapping eQTL interactions with GC treatment below). We used linear regression to remove the effects of these potential confounders at each gene, applied separately in each treatment, and confounder-corrected data were used in all subsequent analyses. We also applied principal component analysis (PCA) to the covariate-corrected data to identify the effects of potentially unmeasured covariates contributing to heterogeneity in the data. As we found that controlling for these principal components had virtually no effect on Bayes factors from our eQTL mapping experiment, we did not regress out the effect of any principal components.

Quality control of expression data. We found that expression levels were highly reproducible across technical and biological replicates. Expression levels between replicate hybridizations were highly correlated (Pearson's $r > 0.95$ between all pairs of replicates – Supplementary Fig. 7a). One array was found to be an outlier, based on all between-array correlations, and was removed from downstream analyses. We found that expression measurements were extremely reproducible for the four LCLs with biological replicates (Pearson's $r > 0.99$ between all pairs of replicates – Supplementary Fig. 7b and 7c). Additionally, we found that the transcriptional response to GC treatment, represented by the log-fold change in expression in GC- over control-treated samples at the 4,568 most responsive genes, was highly replicable across biological replicates, despite replicates being thawed, grown and treated two months apart (Pearson's $r > 0.77$ between all pairs of replicates – Supplementary Fig. 7d).

Identification of differentially expressed genes

Testing for differential expression. In order to identify genes that, on average across individuals, changed expression levels upon treatment with GCs, we performed multiple linear regression at each gene with treatment as the covariate of interest while taking other measured covariates into account. To reduce the effects of outliers, microarray intensity values were quantile normalized to a *N(0,1)* distribution across all samples (treated and untreated). We used the distribution of p-values observed when sample labels are permuted (ten permutations were used), an empirical estimate of the p-value distribution under the null, to estimate the false discovery rate (FDR). Using this approach we found 4,568 differentially expressed genes at an FDR<0.01, including up- and down-regulated genes (Supplementary Fig. 1a). We used the online tool DAVID [7,8] to identify biological categories enriched among up and down-regulated genes, using all genes expressed in LCLs (based on microarray data) as a background.

Traditional tests for differential expression (e.g. a t-test) look for changes in expression that are consistent across individuals, biasing against genes where the response to GCs varies across individuals (e.g. due to genetic variation). Consequently, we did not restrict the analyses of expression variation to the 4,568 differentially expressed genes above.

Sub-sampling experiments to assess relationship between samples size and differential expression results. The larger number of differentially expressed genes we find relative to previous studies may be explained by increased power from our larger sample size. To test this hypothesis, we generated random sub-samples of varying sizes and repeated tests for differential expression. We found a clear relationship between sample size and the number of differentially expressed genes identified (Supplementary Fig. 1b). We found that increasing sample size allowed genes with more variable responses between individuals to be detected more readily (Supplementary Fig. 1c). We also found that increases in sample size allow us to more readily identify target genes with small but consistent changes in expression (Supplementary Fig. 1d). A similar pattern was observed in a survey of gene expression response in soy with a large sample size [9]; thus, it is possible that the number of genes involved in many cellular responses to stimuli is generally underestimated.

Comparison with differential expression in osteoblasts. We compared differential expression results in LCLs to published results from a similar study in osteoblasts[10]. We limited our comparison to the 8,646 genes identified as "expressed" in both tissues. For each of the 3,018 genes we identified as differentially expressed ($p < 0.003$ and $FDR < 0.01$) and that were expressed in osteoblasts, we tested for marginal evidence of differential expression in the osteoblast data ($p < 0.05$). We then applied a one-tailed Fisher's exact test to test the statistical significance of this overlap.

Genetic association mapping

SNP imputation. We used all HapMap SNPs for all mapping experiments described. As TSI LCLs were only typed for phase III SNPs, we used the CEU population sample to impute genotypes at all HapMap phase I and II SNPs. Similarly, we imputed SNPs for phase III YRI LCLs based on the YRI LCLs included in phase I and II. Imputation was performed using BIMBAM [11], which infers missing genotypes based on correlations between missing and typed genotypes observed in samples where all genotypes are typed. QTL mapping results were not qualitatively different if using imputed or genotyped SNPs.

Frequentist association mapping of log-fold change in expression. We tested for association between all HapMap SNPs and transcriptional response at each gene, using log fold change in expression (GC-treated over control-treated expression) as a measure of response. For our candidate gene-based scan for trans-acting eQTLs that influenced response, we tested all HapMap SNPs within 500kb and 100kb (in two separate sets of analyses) of genes encoding the GR and transcription factors that interact with the GR. Interacting transcription factors include the genes that encode the components of the NFkB complex, AP1, Oct1, Oct2, CREB, ETS1, STAT3, STAT5,STAT6, C/EBP, TFIID, T-bet, PU.1/Spi-1, Smad3, Smad4, Smad6, COUP-TFII, IRF3, STIP1, Hic5/Ara55, and nTrip6 (PMID: 17689856). P-values calculated with permutated genotype labels were used as an empirical null distribution. In roder to maintain the correlation structure across genes, the same permutation seed was used for all genes in both candidate gene tests and the genome-wide scan. Ten permutations were performed for the test of variation within 500kb, 100 permutations were used for the test of variation within 100kb and 3 permutations were used for the genome-wide scan. For mapping log fold change at SNPs within 500kb or 100kb of each gene, permutation seeds were set separately at each gene. Association tests were performed using a combination of Python, the R statistical package and the genetic association mapping program PLINK.

Bayesian regression for identifying genetic associations and interaction with treatment

Bayes Factors calculation. We developed a novel Bayesian statistical framework for genetic association analysis in settings where measurements are available on the same individuals in two different conditions (in our case, GC-treated and control-treated). Our methods extend and improve the methods from Barber et al. (2009) to explicitly consider "qualitative interaction" models where genetic variants are associated with measurements in only one of the two conditions. Our method takes into account both sample pairing and the intra-individual correlation of measurements under the two conditions. Software implementing the methods will be made available at http:/stephenslab.uchicago.edu/software.html.

Consider a phenotype measured under two conditions (e.g. gene expression in the presence and absence of GCs) in a sample of size n. We use Y to denote the collection of phenotypic measures, where $Y_1 = (y_{11}...y_{n1})$ represents the phenotypic measurements for n individuals under condition 1 and $Y_2 = (y_{12}...y_{n2})$ are the phenotypic measurements for n individuals under condition 2. In practice we quantile normalize our phenotypic measurements, so Y_1 and Y_2 each follow a standard normal distribution. The relationship between genotype at a given polymorphism $G = (g_1...g_n)$, coded as 0, 1, or 2 copies of the minor allele, and Y is modeled as a mixture of the mean expression in each condition $\mu = (\mu_1, \mu_2)$, the genotypic effects $\beta = (\beta_1, \beta_2)$, and the error term $\varepsilon = (\varepsilon_1, \varepsilon_2)$, which is $N_2(0,V)$ with V representing the 2x2 covariance matrix (thereby capturing correlation between phenotypic measurements in the two conditions):

$$\begin{pmatrix} y_{i1} \\ y_{i2} \end{pmatrix} = \begin{pmatrix} \mu_1 \\ \mu_2 \end{pmatrix} + \begin{pmatrix} \beta_1 g_{i1} \\ \beta_2 g_{i2} \end{pmatrix} + \begin{pmatrix} \varepsilon_{i1} \\ \varepsilon_{i2} \end{pmatrix}$$

We consider the following five different models relating genotype to phenotype across conditions:

0) Null model: $\beta_1 = \beta_2 = 0$
1) No-interaction eQTL model: $\beta_1 = \beta_2 \neq 0$
2) GC-only eQTL model: $\beta_1 \neq 0$ and $\beta_2 = 0$
3) Control-only eQTL model: $\beta_1 = 0$ and $\beta_2 \neq 0$
4) General interaction eQTL model: $\beta_1 \neq 0$ and $\beta_2 \neq 0$ but $\beta_1 \neq \beta_2$

Note that we are defining an interaction between genotype and treatment to be where the genotypic effect is different in the two treatments. Thus models 2-4 involve an interaction, whereas 0 and 1 do not. Models 2 and 3 are sometimes referred to as "qualitative" interactions, whereas model 4 is referred to as a "quantitative" interaction. One important feature of qualitative interactions is that, unlike quantitative interactions, their definition does not depend on the scale of measurement of each phenotype.

The Bayes factor for model *C* is defined as the ratio of the marginal likelihood under model *C* versus the null model. We use $p_C(\,)$ to denote probability distributions under model *C*, and as shorthand for the marginal distribution under model C, so $P^c = p_C(Y)$.

The BFs for models 1-4 all have a similar analytic form, and before describing the assumptions that lead to these BFs we first state this form. Specifically, they have the form:

$$BF(Y, X_0, X_1; \sigma_a) := \left[\sigma_a^{-2} \frac{|X_0' X_0|}{|X_1' X_1 + K_1|}\right]^{d/2} \left[\frac{|RSS(Y|X_0, K_0)|}{|RSS(Y|X_1, K_1)|}\right]^{(n+d-1)/2} \quad (1)$$

where Y is an $n \times d$ matrix, X_0 is an $n \times p$ matrix, X_1 is an $n \times (p+1)$ matrix, and σ_a is a scalar hyper-parameter, all of which will be defined below. In Equation (1), K_0 denotes the $p \times p$ matrix of all 0s; K_1 denotes the $p+1 \times p+1$ matrix with σ_a^{-2} at position $(p+1, p+1)$ and zeros elsewhere, and:

$$RSS(Y|X, K) := Y'Y - Y'X(X'X + K)^{-1} X'Y$$

is a Bayesian analogue of the standard residual sums of squares matrix from regressing Y on X. (Note that A' denotes the transpose of a matrix A.) A brief note on the intuition behind this expression: effectively (1) compares the fit of a regression of Y on X_0 with the regression of Y on X_1 (e.g. look at the ratio of the sums of squares terms). In particular, the BF will be big if the residuals sums of squares matrix from regressing Y on X_1 is much smaller (as measured by its determinant) than obtained from regressing Y on X_0.

Under the assumptions we make (see below), the BFs for models 1-4 have the form given by (1), but with different values for Y, X_0, and X_1. We now turn to describing the assumptions that lead to this form, and the specific values of Y, X_0, and X_1 for each model.

First we describe the prior distributions, and the resulting BF, for model 4 vs. the null (i.e. BF^4). Under the null we assume standard conjugate priors for μ and V:

$$V \sim InverseWishart\,(m, \Psi)$$

$$\mu | V \sim N_2(0, \sigma_\mu V)$$

where N_2 denotes the bivariate normal distribution. Under model 4 we must also specify a prior distribution for β, and again we use the standard conjugate prior:

$$\beta | V \sim N_2(0, \sigma_a V)$$

These priors are computationally convenient because they lead to BFs that are analytically tractable. To deal with the hyperparameters m, Ψ, and σ_μ, we take the limits $m \to 1$, $\Psi \to 0$, $\sigma_\mu \to \infty$. We choose these limits because the resulting BF^4 has a number of attractive properties: for example, it is invariant to changes in the mean and variance of the phenotypes Y, and, under further conditions on σ_a, is monotonically increasing with the standard likelihood ratio test of model 4 vs. the null (M Stephens, unpublished data). The hyperparameter σ_a controls the typical size of the effects (β); since this is unknown in practice. Under these priors, the marginal likelihoods, P^4 and P^0, can be computed analytically and (in the limits described above), $BF^4 = P^4 / P^0$, has the form (1), with $Y = [Y_1, Y_2]$ (so d = 2); $X_0 = [1]$ (where 1 is a column vector of all 1s); X_1 = [1; g] (where g is a column vector of the genotypes at a SNP of interest, coded as 0, 1 or 2 copies of a reference allele). Note that, following the intuitive interpretation of the BF (1) noted above, this BF will be large if the regression of Y on an intercept and genotype (X_1) is much better than the regression of Y on an intercept alone (X_0).

Next we describe the Bayes factor for model 2, BF^2. Under model 2, Y_1 is independent of genotype, so the model can be written $p_2(Y_1, Y_2 | g) = p_2(Y_1) p_2(Y_2 | Y_1, g)$. We make the additional assumptions that i) $p_2(Y_1)$, which does not depend on genotype, is the same as $p_0(Y_1)$; ii) $p_2(Y_2 | Y_1, g)$ is the same as $p_4(Y_2 | Y_1, g)$. Under these assumptions, the BF for model 2, $BF^2 = P^2 / P^0$, is given by (1) with $Y = [Y_2]$ (so d = 1), $X_0 = [1, Y_1]$; and $X_1 = [1, Y_1, g]$ Here the intuition is that this BF will be large if the regression of Y_2 on an intercept and Y_1 and a genotype (X_1) is much better than the regression of Y_2 on an intercept and Y_1 alone (X_0). That is, it will be large if Y_2 is associated with genotype controlling for Y_1.

Under model 3, Y_2 is independent of genotype, and following the same logic as for model 2 the BF, $BF^3 = P^3 / P^0$, is given by (1) with $Y = [Y_1]$, $X_0 = [1, Y_2]$, and $X_1 = [1, Y_2, g]$.

To compute a BF for model 1, we use the transformation $Z_1 := Y_1 - Y_2$, $Z_2 := Y_1 + Y_2$ (note that $p_1(Y_1, Y_2 | g) / p_0(Y_1, Y_2) = p_1(Z_1, Z_2 | g) / p_0(Z_1, Z_2)$ because the Jacobian term that occurs due to the transformation cancels out in the numerator and denominator). Further, under model 1, genotype g has the same effect on both Y_1 and Y_2, so it is independent of Z_1, and we can write $p_2(Z_1, Z_2 | g) = p_2(Z_1) p_2(Z_2 | Z_1, g)$. Again, assuming that $p_1(Z_1) = p_0(Z_1)$ and $p_1(Z_2 | Z_1, g) = p_2(Z_2 | Z_1, g)$, the Bayes Factor for model 1, $BF^1 = P^1 / P^0$, has the form (1) with $Y = [Z_2]$, $X_0 = [1, Z_1]$, and $X_1 = [1, Z_1, g]$.

The hyperparameter σ_a. The hyperparameter σ_a controls the expected effect sizes. As σ_a is unknown, we assume a prior distribution with weights $W = (w_1 ... w_J)$ on distinct values $\sigma_a = (\sigma_a^1 ... \sigma_a^J) = (0.8, 1.0, 1.2, 1.6)$. Under these prior assumptions, the Bayes factor for each SNP is a weighted average of the BFs calculated under each value of σ_a as follows:

$$BF^C = \sum_{j=1}^{J} w_j BF^C(\sigma_a^j)$$

where $BF^C(\sigma_a^j)$ is the Bayes factor for model *C* calculated assuming $\sigma_a = \sigma_a^j$. Effectively, this replaces the hyperparameter σ_a with another set of hyperparameters $W = (w_1 ... w_J)$. For gene expression experiments, these hyperparameters are estimated from the data across all genes, using the hierarchical model described below. For secretion experiments we fixed the weights to all be equal, since we judged that the small number of assayed proteins would not suffice to estimate these weights reliably.

Combining information across SNPs. The above description of Bayes factor calculations considers the relationship between one polymorphism (e.g. SNP) and one phenotype (e.g. gene expression) measured under two conditions. In what follows we use BF_{ki}^C to denote this Bayes factor computed for SNP *i* in gene *k*. We now consider combining these BFs for all SNPs within 500kb of the gene to compute an overall gene-wide BF, BF_k^C, that measures the overall evidence that gene *k* is affected by an eQTL of model *C* (i.e. that gene *k* follows model *C*). Specifically, we define this gene-wide BF to be equal to the ratio P_k^C / P_k^0 where P_k^C is the marginal likelihood under a model where gene *k* contains exactly one SNP affecting expression (we refer to this SNP as an eQTN) according to model *C* (with all SNPs equally likely) and P_k^0 is the marginal likelihood under the null model that no SNP affects expression at gene *k*. Under this model the gene-wide BF is simply the average of BF_{ki}^C across all S_k SNPs:

$$BF_k^C = P_k^C / P_k^0 = \frac{1}{S_k} \sum_{i=1}^{S_k} BF_{ki}^C$$

(We note that if a gene actually contains multiple eQTNs our approach, which assumes each gene contains at most one eQTN, will tend to assign that gene to the model of the strongest eQTN. This could cause us to miss interaction eQTNs at genes that also contain strong non-interaction eQTNs.)

Hierarchical model. To combine information across all genes we use a hierarchical model (e.g. Veyrieras et al 2007) in which the parameters are: the proportion of genes containing eQTNs following each of the models described above ($\Pi = (\pi_0, \pi_1, \pi_2, \pi_3, \pi_4)$) and the weights $W = (w_1 ... w_g)$ in the prior for σ_a described above. Specifically, in this hierarchical model the probability of the expression data Y_k for gene *k*, given parameters (Π, W), is

$$P(Y_k \mid \Pi, W) = \pi_0 P_k^0 + \pi_1 P_k^1 + \pi_2 P_k^2 + \pi_3 P_k^3 + \pi_4 P_k^4$$

$$= P_k^0 (\pi_0 + \pi_1 BF_k^1 + \pi_2 BF_k^2 + \pi_3 BF_k^3 + \pi_4 BF_k^4)$$

Assuming all genes are independent given these parameters, the overall likelihood is given by:

$$L(\Pi, W \mid Y) = \prod_k P(Y_k \mid \Pi, W, X)$$

We used an EM-algorithm to obtain maximum likelihood estimates of hyperparameters, namely $(\hat{\Pi}, \hat{W})$. We also constructed the 95% profile-likelihood confidence intervals for all estimated parameters. Estimated proportions of genes under each model, with confidence intervals, are shown for all reported applications of this method in Supplementary Table 1.

Posterior probabilities and Bayesian estimate of false discovery rate. We calculated the posterior probability that gene *k* follows model *C* using:

$$P(gene\ k\ \ follows\ C \mid Y, \hat{\Pi}, \hat{W}) = \frac{\hat{\pi}_c BF_k^C}{\sum_{c=0}^{4} \hat{\pi}_c BF_k^c}$$

where BF_k^C is calculated using estimated weights $\hat{W}$.

For each gene, the posterior probability that it harbors an interacting eQTL is the sum of posterior probabilities (as computed above) for models *C*=2, 3, and 4. For any given threshold on this posterior probability (eg 0.7 is used in the text) we estimate the false discovery rate as the mean probability of a false positive (1-posterior probability) across genes exceeding the threshold. (Note that, for interacting eQTLs, we are therefore, appropriately, defining false positives to include not the null model, but also the no-interaction eQTL model).

Comparisons to mapping log-fold change. To assess the power of our method relative to a traditional test for association with response, we compared, at each gene, the evidence of an interaction eQTL from mapping the log-fold change in expression (specifically the minimum p-value) to evidence of an interaction eQTL from the Bayesian regression (posterior probability of an eQTL following one of the interaction models). We found that, at a false discovery rate threshold of 0.10, our method identified more interactions, indicating that our method provides an increase in power over directly mapping the log-fold change (Supplementary Fig. 2).

Combining mapping results across populations. We found that BFs (calculated for each SNP and gene comparison) often differed depending on the population used for eQTL mapping. These discrepancies were associated with differences in heterozygosity between populations (p-value = 1.2×10^{-161}, r^2 = 0.054), suggesting that differences may be due, in part, to differences in allele

frequency at eQTLs. To combine information across populations, previous cis eQTL mapping studies have quantile normalized phenotypes within populations and then pooled multiple populations together [12]. This approach could reduce power, however, if there are substantial differences in allele frequency at eQTLs. To avoid this issue, we chose to calculate BFs for each SNP-gene comparison separately in the two populations and then multiply the BFs together to combine information across populations.

It is important to note, however, that multiplying Bayes factors across populations ignores the direction of genotypic effects in the two populations. To assess the reproducibility of our mapping results across populations, we used linear regression to estimate the genotypic effect on expression of each eQTL that interacted with GC treatment separately in TSI and YRI. We used the most likely model to identify the relevant treatment condition for each eQTL's genotypic effect, and regressed expression in that condition on genotype at the most likely eQTN while controlling for expression in the opposite treatment condition. For example, the genotypic effect of GC-only eQTLs was estimated by regressing expression in GC-treated samples on genotype with expression in control-treated samples as a covariate. We find that genotypic effects are largely consistent across populations. Specifically, we find that, excluding eQTNs with minor allele frequencies less than 0.05, 80% of the eQTNs that interacted with GC treatment had concordant genotypic effects on log-fold change across populations. We found that GC-only eQTNs were more concordant, where 89% of GC-only and only 67% of control-only eQTNs (posterior probability > 0.7) had effects in the same direction in both populations. eQTNs with effects in opposite directions across populations are noted in Supplementary Table 2.

Minor allele frequencies for eQTLs. We also compared the distribution of minor allele frequencies of the candidate eQTN was between the three eQTL models. We did not observe any significant differences (Supplementary Fig. 9).

Assaying allelic imbalance using quantitative real-time PCR

Cis-acting regulatory polymorphisms will cause differential expression between chromosomes in heterozygotes. On a population level, this will cause unequal representation of alleles at coding polymorphisms on the same haplotype in the mRNA of individuals heterozygous for the regulatory polymorphism. We used TaqMan quantitative genotyping assays to assay allelic imbalance at coding SNPs in LD with eQTLs that interacted with GC treatment. Imbalanced expression of the two coding alleles is an independent line of evidence for a cis-acting regulatory polymorphism and for the configuration of the effect in the two treatment conditions (i.e. the interaction model).

Total RNA from an aliquot of the same culture samples used to hybridize microarrays (this was a separate RNA extraction as that used to hybridize microarrays) was synthesized into cDNA using the High-Capacity cDNA Reverse Transcription Kit (Applied Biosystems, Foster City, CA) according to the manufacturer's protocol. Quantitative real-time PCR was performed in 384 well plates using the ABI PRISM 7900HT Sequence Detection System. The 10 μl reactions contained 5 μl of Taqman® Universal PCR Master Mix with no AmpErase® UNG, 0.25 μl of Taqman® 40X SNP Genotyping Assay, 0.25 μl of nuclease-free water, and 4.5 μl of cDNA. Taqman® 40X SNP Genotyping Assays were purchased from Applied Biosystems. Applied Biosystems standard recommended PCR cycling conditions were used. Raw data were analyzed using ABI SDS software version 2.2.2 (Applied Biosystems, Foster City, CA). To account for differences between the two fluorochromes, a standard curve was built for each of the two alleles using serial dilutions of a genomic DNA from an individual that was heterozygous at the coding SNP. PCR products were quantified for each allele separately in each reaction. For each assay, we calculated the natural log-ratio between the two different alleles. The numerator of this ratio was always the allele associated with increased expression in the corresponding treatment condition. Within each treatment, we quantile normalized allelic log-ratios and used a one-tailed t-test to identify significant differences in average allelic log-ratios between heterozygotes and homozygotes (as an empirical null distribution of allelic log-ratios) at the eQTL.

Assessing population differences in response

Identifying interactions between ancestry and GC treatment. To identify genes that may contribute to inter-ethnic differences in phenotypes related to GCs, we contrasted the transcriptional response to GCs between YRI and TSI LCLs. Differences in transcriptional response between populations will result in differences in average expression levels that differ depending on treatment, as opposed to GC-independent population differences that will be identical in both treatments. As this is analogous to gene-environment interactions, we used the same statistical framework to identify genes with differences in transcriptional response between populations. Covariate-corrected expression levels were quantile normalized across individuals (both YRI and TSI) for each gene to reduce the effect of outliers. As population differences at the phenotypic level may reflect population differences in response following a consistent pattern across many genes, we identified the direction of population differences at each gene in terms of log-fold change and found that up-regulated genes tended to show a stronger response in YRI LCLs (Supplementary Fig. 3).

Expected population differences based on eQTLs. Differences in allele frequency at QTLs will cause differences in mean phenotypic values between populations. Concordance between allele frequency differences and differences in phenotypic means can be used to validate QTL effects. This is because allele frequency differences at false positive QTLs will not cause differences in phenotypic means between populations. As a validation of the genetic effects on the transcriptional response to GCs at the cis eQTLs, we asked if allelic differentiation was consistent with observed differences between populations in average transcriptional response (i.e. log-fold change) at corresponding genes.

For a phenotype affected by a biallelic polymorphism, the mean phenotypic value for a population (μ) is expected to be the sum of the mean phenotypic values in each genotype class (genotypes coded by copies of minor allele (μ_0, μ_1, μ_2)) weighted by their frequencies (f_0, f_1, f_2):

$$\mu = f_0\mu_0 + f_1\mu_1 + f_2\mu_2$$

Assuming Hardy-Weinberg equilibrium, genotype frequencies can be expressed as a function of major allele frequency (p = frequency of major allele):

$$\mu = p^2\mu_0 + 2p(1-p)\mu_1 + (1-p)^2\mu_2$$

Assuming additive genotypic effects allows us to express the mean phenotypic value per genotype class in terms of the genotypic effect (β).

$$\mu = p^2\mu_0 + 2p(1-p)(\mu_0 + \beta) + (1-p)^2(\mu_0 + 2\beta)$$

This reduces, algebraically, to:

$$\mu = \mu_0 + 2\beta + 2\beta p$$

Assuming that the mean phenotypic value was the same in both populations (within each genotype class) and that the genotypic effect was the same, the predicted difference in mean phenotypic value between two populations (e.g. YRI and TSI) can, therefore, be expressed as follows:

$$predicted\ difference = \mu_{YRI} - \mu_{TSI} = (\mu_0 + 2\beta + 2\beta p_{YRI}) - (\mu_0 + 2\beta + 2\beta p_{TSI}) = 2\beta(p_{YRI} - p_{TSI})$$

We compared observed differences between populations to expectations based on the allele frequencies and the genotypic effects at eQTLs (see Figure 3a).

References

1. Akey, J.M., Biswas, S., Leek, J.T. & Storey, J.D. On the design and analysis of gene expression studies in human populations. *Nat Genet* **39**, 807-8; author reply 808-9 (2007).
2. Du, P., Kibbe, W.A. & Lin, S.M. lumi: a pipeline for processing Illumina microarray. *Bioinformatics* **24**, 1547-8 (2008).
3. Lin, S.M., Du, P., Huber, W. & Kibbe, W.A. Model-based variance-stabilizing transformation for Illumina microarray data. *Nucleic Acids Res* **36**, e11 (2008).
4. Dunning, M.J., Ritchie, M.E., Barbosa-Morais, N.L., Tavare, S. & Lynch, A.G. Spike-in validation of an Illumina-specific variance-stabilizing transformation. *BMC Res Notes* **1**, 18 (2008).
5. Stark, A.L. et al. Heritable and non-genetic factors as variables of pharmacologic phenotypes in lymphoblastoid cell lines. *Pharmacogenomics J* (2010).
6. Choy, E. et al. Genetic analysis of human traits in vitro: drug response and gene expression in lymphoblastoid cell lines. *PLoS Genet* **4**, e1000287 (2008).
7. Huang da, W., Sherman, B.T. & Lempicki, R.A. Systematic and integrative analysis of large gene lists using DAVID bioinformatics resources. *Nat Protoc* **4**, 44-57 (2009).
8. Dennis, G., Jr. et al. DAVID: Database for Annotation, Visualization, and Integrated Discovery. *Genome Biol* **4**, P3 (2003).
9. Zhou, L. et al. Infection and genotype remodel the entire soybean transcriptome. *BMC Genomics* **10**, 49 (2009).
10. Grundberg, E. et al. Global analysis of the impact of environmental perturbation on cis-regulation of gene expression. *PLoS Genet* **7**, e1001279 (2011).
11. Guan, Y. & Stephens, M. Practical issues in imputation-based association mapping. *PLoS Genet* **4**, e1000279 (2008).
12. Veyrieras, J.B. et al. High-resolution mapping of expression-QTLs yields insight into human gene regulation. *PLoS Genet* **4**, e1000214 (2008).
